# Neuroinflammation in Autoimmune Disease and Primary Brain Tumors: The Quest for Striking the Right Balance

**DOI:** 10.3389/fncel.2021.716947

**Published:** 2021-08-13

**Authors:** Dana Mitchell, Jack Shireman, Elizabeth A. Sierra Potchanant, Montserrat Lara-Velazquez, Mahua Dey

**Affiliations:** ^1^Department of Pediatrics, Indiana University School of Medicine, Indianapolis, IN, United States; ^2^Dey Malignant Brain Tumor Laboratory, Department of Neurological Surgery, University of Wisconsin School of Medicine and Public Health, Madison, WI, United States

**Keywords:** brain tumor, glioma, neuroinflammation, multiple sclerosis, glioblastoma, immunotherapy, immune privileged, autoimmune

## Abstract

According to classical dogma, the central nervous system (CNS) is defined as an immune privileged space. The basis of this theory was rooted in an incomplete understanding of the CNS microenvironment, however, recent advances such as the identification of resident dendritic cells (DC) in the brain and the presence of CNS lymphatics have deepened our understanding of the neuro-immune axis and revolutionized the field of neuroimmunology. It is now understood that many pathological conditions induce an immune response in the CNS, and that in many ways, the CNS is an immunologically distinct organ. Hyperactivity of neuro-immune axis can lead to primary neuroinflammatory diseases such as multiple sclerosis and antibody-mediated encephalitis, whereas immunosuppressive mechanisms promote the development and survival of primary brain tumors. On the therapeutic front, attempts are being made to target CNS pathologies using various forms of immunotherapy. One of the most actively investigated areas of CNS immunotherapy is for the treatment of glioblastoma (GBM), the most common primary brain tumor in adults. In this review, we provide an up to date overview of the neuro-immune axis in steady state and discuss the mechanisms underlying neuroinflammation in autoimmune neuroinflammatory disease as well as in the development and progression of brain tumors. In addition, we detail the current understanding of the interactions that characterize the primary brain tumor microenvironment and the implications of the neuro-immune axis on the development of successful therapeutic strategies for the treatment of CNS malignancies.

## Introduction

Inflammation is an adaptive response to infection or tissue injury that arises secondary to the activation of multifactorial networks comprising the innate and adaptive immune systems (Medzhitov, 2008). While historically highlighted for its role as a protector against invading pathogens, based on recent literature, it is now more appropriate to recognize inflammation as a response to any insult that disrupts normal tissue homeostasis (Medzhitov, 2008). The role of inflammation in defending against pathogens, responding to injury, tumor surveillance and maintaining homeostasis has been widely studied in the periphery. However, elucidating its contribution to central nervous system (CNS) homeostasis and, to some extent CNS pathologies, has lagged behind. This gap in knowledge is in part due to the previously held belief based on early experiments from mid 1900s (Medawar, 1948; Galea et al., 2007), that the CNS represented an immune-privileged environment protected behind an impenetrable blood-brain-barrier (BBB), devoid of any CNS resident professional antigen presenting cells (APC), with low expression of immune recognition molecules [e.g., major histocompatibility class (MHC- I and II) and the absence of classical lymphatic drainage system, Louveau et al., 2015a].

Since these initial studies, there has been significant advancement in our understanding of CNS micro-anatomy, physiology and immunology. Animal studies have clearly demonstrated that there are resident APCs in mouse brain (Dey et al., 2015) as well as the presence of a fully functional lymphatic system that lies within the meninges and drains into deep cervical lymph nodes (Andres et al., 1987; Aspelund et al., 2015; Louveau et al., 2015b) where CNS antigens have been shown to induce an immune response (Cserr et al., 1992). Together these studies have challenged the foundation of the old “immune privilege” dogma. A large body of recent literature suggests that inflammatory responses play a critical role in the maintenance of CNS homeostasis and response to injury (Gadani et al., 2015). However, immune responses in the CNS can have both beneficial or detrimental effects on CNS functionality based on the specific context in which they occur. Under physiologic conditions, transient inflammatory responses serve to facilitate CNS function, promote healing, and re-establish a homeostatic state (Coussens and Werb, 2002), while dysregulation of this response can result in exaggerated or chronic inflammatory states that potentiate disease pathogenesis (Coussens and Werb, 2002; Medzhitov, 2008). Within the CNS, pathological inflammation is associated with a wide range of conditions, including autoimmune neuroinflammatory disorders such as multiple sclerosis (MS) and antibody-mediated encephalitis (Ransohoff and Engelhardt, 2012; Dalmau and Graus, 2018; Brambilla, 2019). Furthermore, chronic inflammation or immune evasion is also known to drive tumorigenesis, and the same mechanisms that serve to maintain and restore CNS homeostasis can be hijacked to promote the initiation and propagation of primary CNS tumors such as glioblastoma (GBM) (Wang et al., 2018; Brandao et al., 2019; Piperi et al., 2019).

Despite their obvious differences, cancer and autoimmune disease share a similar fundamental mechanism of pathogenesis in which there is an excessive proliferation of pathogenic cells (e.g., tumor cells and autoreactive immune cells, respectively) occurring in combination with both local and systemic, chronic immune system dysfunction. In both settings, the scales have been tilted just enough to elicit such severe and chronic dysregulation that the physiologic regulatory mechanisms become incapable of restoring immunological balance. On a basic, fundamental level these two pathologies can be thought to represent the opposite extremes of the same spectrum, where on one end exists neuro-immune hyperactivity and on the other, suppression. However, the premise that these pathologies are characterized by polarization of the neuro-immune axis to either completely hypo- or hyper-activity is far too simplistic. Rather, the significant cellular heterogeneity, cellular plasticity, mechanistic non-redundancy (multiple, distinct mechanisms capable of producing same outcome) and functional redundancy (single mechanisms capable of producing multiple, distinct outcomes) of the neuro-immune axis permits for dynamic, context-specific responses that evolve over the course of both disease states. As such, the information gleaned from the study of autoimmune neuroinflammatory disorders may provide critical insight into how to most effectively manipulate the neuro-immune axis to mount an efficient anti-tumor response for the treatment of primary brain tumors such as GBM. Conversely, understanding how GBM so eloquently drives and maintains such profound immunosuppression in both the CNS and systemically may facilitate the development of treatments for autoimmune neuroinflammatory disorders.

In this review, we provide an overview of the neuro-immune axis in steady state and the mechanisms underlying chronic neuroinflammation in autoimmune neuroinflammatory disease as well as in the development and progression of primary brain tumors. While it should be noted that the neuro-immune axis plays an important role in a wide variety of CNS pathologies including, neurodegenerative diseases, metastasis and the spectrum of primary brain tumors, in this review, we highlight the complexities and context-specificity of neuro-immune axis disruption by focusing specifically on multiple sclerosis (MS) and glioblastoma, the most common neuroinflammatory disorder and primary brain tumor in adults, respectively. In addition, we detail the implications of therapeutically modulating neuro-immune axis in the development of successful immunotherapy for the treatment of CNS malignancies and autoimmune neuroinflammatory disorders by focusing on immunotherapeutic developments in the treatment of these two disease states.

## Neuro-Immune Surveillance and CNS Steady State

**FIGURE 1:**
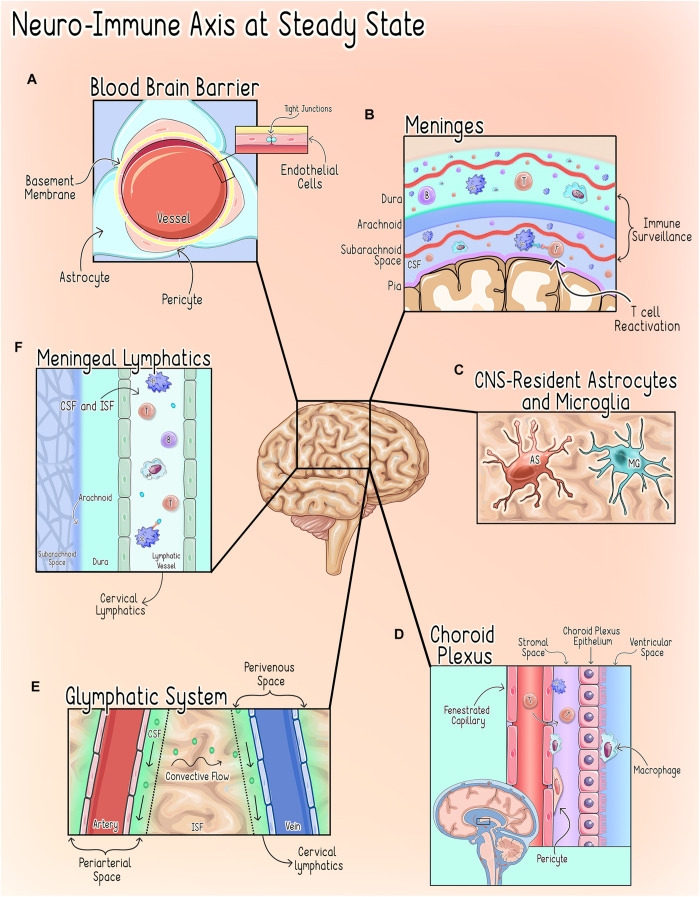
Neuro-Immune axisat steady state. CNShomeostasis is maintained by a highly regulated and compartmentalized system of anatomical, cellular, and chemical components (Galea and Perry, 2018). **(A)** The Blood Brain Barrier (BBB). The BBB refers to the unique properties of the cerebral vessels, which are formed by endothelial cells (ECs) characterized by continuous intracellular tight junctions, a lack of fenestrations, and low expression of leukocyte adhesion molecules and supported by interactions with pericytes and astrocytic end-feet (Abbott et al., 2006; Ransohoff and Engelhardt, 2012). Together these properties severely restrict access to the CNS parenchyma through the cerebral vasculature (Abbott et al., 2006; Ransohoff and Engelhardt, 2012). **(B)** The Meninges. The meninges are a delicate three-layer structure consisting of the dura, arachnoid and pia mater. In addition to grossly isolating the CNS from the periphery, the meninges are an immunologically active organ that play an important role in CNS immune surveillance by allowing peripheral immune cells to sample brain-derived antigens without directly accessing the parenchyma during homeostatic states (Engelhardt and Ransohoff, 2005; Siffrin et al., 2009; Mundt et al., 2019; Frederick and Louveau, 2020). Macrophages are thought to be the most abundant immune cell in murine meninges during steady-state, but populations of T-cells, B-cells, plasma cells (PCs), dendritic cells (DCs), monocytes, neutrophils, natural killer (NK) cells, and γδ T-cells have also been identified (Radjavi et al., 2014; Van Hove et al., 2019; Fitzpatrick et al., 2020). Both CNS antigen (Ag)-specific and non-CNS antigen (Ag)-specific T-cells can accumulate in the meningeal and perivascular spaces, however, non-CNS Ag-specific T-cells are not reactivated by dendritic cells upon entering the CNS and thus do not invade further into the brain parenchyma (Bartholomäus et al., 2009; Mues et al., 2013; Schläger et al., 2016). This process of T-cell reactivation at the CNS interface is a critical step in regulating T-cell access to the parenchymal compartment (Mundt et al., 2019). **(C)** CNS-Resident Microglia and Astrocytes. CNS-Resident Microglia and Astrocytes serve as the first responders to disruptions of homeostasis and, upon activation, initiate a transient, self-limiting neuroinflammatory response intended to protect the vital structures and functions of the CNS (DiSabato et al., 2016). **(D)** The Choroid Plexus (CP). The choroid plexus, a vascular structure within the ventricular system, serves as the anatomical structure of the blood-CSF barrier (BCSFB) (Wolburg and Paulus, 2010; Demeestere et al., 2015) and under physiologic conditions hosts a diverse population of immune cells and participates in immune surveillance during steady state (Wolburg and Paulus, 2010; Korin et al., 2017; Dixon and Pérez, 2020; Shipley et al., 2020). **(E)** The Glymphatics System. The glymphatics system is a glial-dependent mechanism that relies on the exchange of cerebrospinal fluid (CSF) and interstitial fluid (ISF) to deliver substances across the brain parenchyma and clear CNS waste into systemic circulation (Louveau et al., 2017; Benveniste et al., 2019). CSF flows into the periarterial space from the subarachnoid space (SAS) where it diffuses into the parenchyma (Frederick and Louveau, 2020). This influx of CSF, drives the interstitial fluid (ISF) into the CSF within the perivenous space where it is cleared into the cervical lymph nodes (Alves de Lima et al., 2020; Frederick and Louveau, 2020; Papadopoulos et al., 2020). **(F)** Meningeal Lymphatics. The meningeal lymphatics are located within the dura mater and serve to carry CSF received from the subarachnoid space (SAS) and ISF from the glymphatics system to the cervical lymph nodes (Mascagni, 1787; Lecco, 1953; Aspelund et al., 2015; Louveau et al., 2015b). This process allows for the drainage of immune cells and brain-derived antigens from the CNS to the peripheral lymph nodes (Louveau et al., 2018). T, T cell; B, B cell; DC, Dendritic Cell; Mφ, Macrophage; MG, Microglia; AS, Astrocyte.

The CNS microenvironment is comprised of unique anatomical and cellular components designed to minimize endogenous and exogenous threats that could compromise CNS function. To carry out this elegant task, the CNS microenvironment employs multiple distinct and parallel levels of regulation mediated by several anatomical interfaces that serve both unique and analogous functions as described in Figure 1.

### Key Cellular Components

Although the anatomical interfaces limit access of many neuro toxic chemicals and cellular agents to the CNS parenchyma, there is still baseline immune surveillance and response to injury that occurs within the parenchymal compartment (Galea et al., 2007). This is largely carried out through concerted efforts of two CNS-resident specialized cell populations: microglia and astrocytes (Galea et al., 2007).

### Astrocytes

**FIGURE 2:**
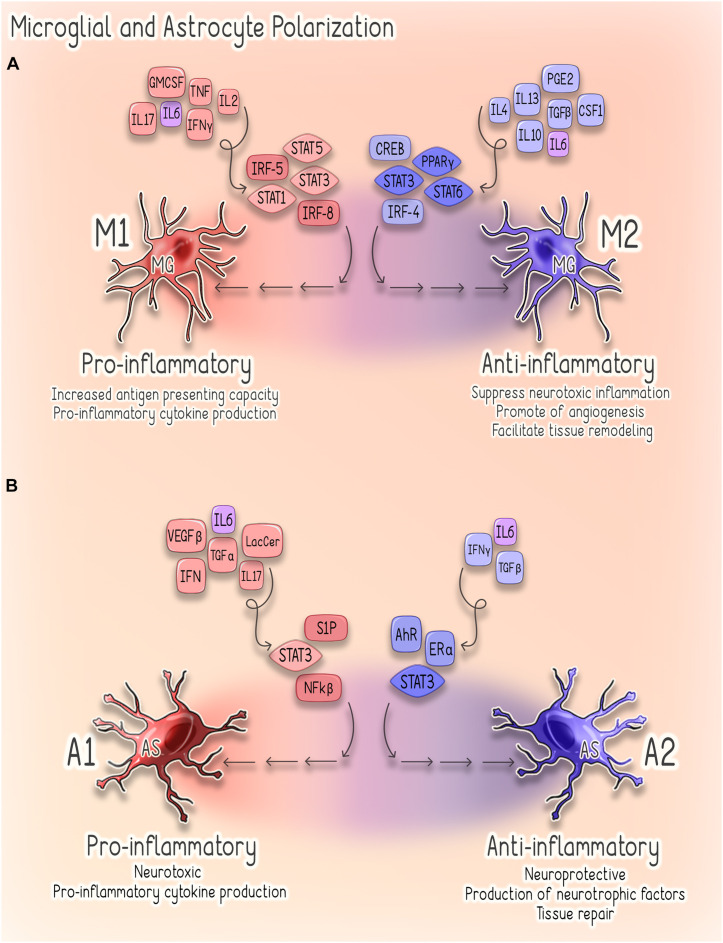
Astrocyte and Microglial Polarization. **(A)** Microglial Polarization. Microglial M1 polarization is thought to be driven predominantly by signal transducer and activator of transcription (STAT) 1/3/5 signaling and interferon regulatory factors 5 (IRF-5) and 8 (IRF-8) (Lawrence and Natoli, 2011; Yan et al., 2018). These M1-activated cells produce pro-inflammatory cytokines (e.g., TNF-α, IL-1β, IL-6, IFNy, and IL-23) and are characterized by increased antigen presenting activity secondary to increased expression of MHC-II and co-stimulatory (CD80 and CD86) molecules (Lawrence and Natoli, 2011; Li and Graeber, 2012). Conversely, M2 polarization depends on STAT3/6 signaling, peroxisome proliferator-activated receptor gamma (PPARγ)-mediated nuclear factor kappa B (NF-kβ) suppression, CREB-C/EBPβ and interferon regulatory factor 4 (IRF4) (Li and Graeber, 2012). Cells exhibiting this phenotype promote tissue repair through the suppression of neurotoxic inflammation as well as through stimulating angiogenesis and tissue remodeling (Li and Graeber, 2012). **(B)** Astrocyte Polarization. Signaling pathways implicated in regulating astroglial activation and determining phenotype, include the NF-kβ, sphingosine 1-phosphate (S1P) receptor, JAK/STAT and type 1 interferon (IFN-I) pathways (Brambilla, 2019). Several factors promote activation of NF-kβ signaling in reactive astrocytes, including glycolipid lactosylceramide (LacCer), type I interferons, TGF-α and VEGF-β (Brambilla, 2019). Activation of NF-kβ by these factors stimulates astrocytes to adopt a pro-inflammatory (A1) (Brambilla et al., 2009). Similarly, estrogen signaling through estrogen receptor alpha (ERα) suppresses NF-kβ signaling and inhibits astrocytes from adopting a pro-inflammatory phenotype (Giraud et al., 2010; Luchetti et al., 2014). Astrocytic IFNy signaling also acts to suppress CNS inflammation through negative regulation of NF-kβ (Rothhammer et al., 2016). Activation of the IFNy pathway upregulates astrocyte expression of the aryl hydrocarbon receptor (AhR), which suppresses inflammation via suppressor of cytokine signaling 2 (SOCS2)-dependent inhibition of NF-kβ (Rothhammer et al., 2016). Conversely, activation of S1P receptor signaling promotes a pro-inflammatory (A1) phenotype in reactive astrocytes (Van Doorn et al., 2010; Choi et al., 2011; Rothhammer et al., 2017). STAT3 signaling can drive astrocytes to adopt either pro- or anti-inflammatory phenotypes in a highly context-specific manner (Ceyzériat et al., 2016). A1 astrocytes are characterized by a pro-inflammatory, neurotoxic phenotype, whereas A2 astrocytes are anti-inflammatory, neuroprotective and promote tissue repair (Li et al., 2020).

Astrocytes are the most abundant glial cells in the CNS, and in steady state they perform a wide range of functions that are critical to CNS health (Sofroniew, 2009; Sofroniew and Vinters, 2010; Colombo and Farina, 2016; Linnerbauer et al., 2020). Notably, astrocytes are responsible for the maintenance of extracellular fluid, ion, and neurotransmitter homeostasis, as well as the modulation of synaptic function and remodeling (Sofroniew, 2009; Colombo and Farina, 2016). Astrocytes also play a critical role in supporting the integrity of the BBB and regulating immune cell trafficking within the CNS (Colombo and Farina, 2016; Linnerbauer et al., 2020). Furthermore, astrocytes themselves respond to disruptions of homeostasis through the secretion of cytokines and chemokines and, in response to CNS injury, also undergo a spectrum of heterogeneous morphological and functional changes (Sofroniew, 2009; Linnerbauer et al., 2020). This process, termed reactive astrogliosis, exists on a graded continuum in which the nature, severity, and type of CNS injury influences the outcome and, as such, reactive astrogliosis can have both beneficial and detrimental effects depending on specific context (Sofroniew, 2009, 2015; Colombo and Farina, 2016). Additionally, a recent elegant study conducted in adult mice and correlated with human astrocyte gene signatures suggests the existence of astrocytic subpopulations that demonstrate regional, cellular, molecular, and functional diversity (John Lin et al., 2017). Thus, it is likely a combination of timing, context, and the specific astrocyte subpopulation that determines how these cells contribute to the neuroinflammatory response (Sofroniew, 2009; Linnerbauer et al., 2020). Broadly, reactive astrocytes are classified as either pro-inflammatory/neurotoxic (A1) or anti-inflammatory/neuroprotective (A2) (Sofroniew, 2009, 2015; Sofroniew and Vinters, 2010; Liddelow et al., 2017). While much remains to be elucidated regarding the mechanisms underlying astrocytic phenotypic plasticity, the NF-kβ sphingosine 1-phosphate (S1P) receptor, JAK/STAT, and type 1 interferon (IFN-I) pathways have been implicated in the regulation of astroglial activation (Figure 2; Brambilla, 2019; Linnerbauer et al., 2020).

### Microglia

Microglia, the resident macrophages of the CNS, are characterized by significant plasticity in both phenotype and function, which allows them to serve several critical roles in the maintenance of CNS homeostasis (Butovsky and Weiner, 2018). In resting state, microglia provide the baseline parenchymal immunity by surveilling the CNS for tissue damage and other threats to homeostasis (Suzumura et al., 1993; Colonna and Butovsky, 2017; Butovsky and Weiner, 2018; Prinz et al., 2019; Rodríguez-Gómez et al., 2020; Spittau et al., 2020; Zia et al., 2020). In response to disruptions of homeostasis, microglia were traditionally believed to assume either a pro-inflammatory/neurotoxic (M1) or anti-inflammatory/neuroprotective (M2) phenotype (Mosser and Edwards, 2008; Lawrence and Natoli, 2011; Landry et al., 2020). Recent evidence, however, suggests that the categorization of microglia into such polarized phenotypes is a drastic oversimplification. It is now understood that these phenotypes exist on a spectrum from neurotoxic (M1) to neuroprotective (M2) (Lawrence and Natoli, 2011; Hu et al., 2015). A more recent study in which single microglial cells isolated from multiple anatomical regions within the mouse CNS were analyzed, further identified distinct subpopulations of microglia present during homeostasis (Masuda et al., 2019). These microglial subpopulations are characterized by unique transcriptional programs that exist on a continuum rather than in discrete clusters (Masuda et al., 2019). While the precise molecular determinants responsible for this continuum of functional outcomes have not been completely elucidated, significant advances in our understanding of the transcriptional regulation of microglial polarization have been made as reviewed in Lawrence and Natoli (2011). Important factors and pathways that have been implicated in microglial polarization are illustrated in Figure 2. However, it is important to recognize that the process of microglial polarization is highly context-specific, and the spectrum of observed phenotypes may reflect activation of specific pathways by distinct stimuli, as well as crosstalk between various pathways (Lawrence and Natoli, 2011).

## Neuroinflammation: A Systematic Protective Response or an Uncontrolled Destructive Phenomenon

The post-mitotic nature of the brain’s most vital constituents, the neurons, makes them extraordinarily vulnerable to damage from which recovery would require a significant degree of regenerative capacity. The uniquely protective CNS environment created by anatomical and cellular components serves not as an absolute barrier, but as a dynamic regulatory system that facilitates optimal CNS function through the maintenance of a homeostatic state. Disruptions of homeostasis or manipulation of the neuro-immune axis by pathological processes, however, trigger the adaptive response of neuroinflammation. Neuroinflammation, which is mediated by cytokines, chemokines, and other factors produced by CNS-resident as well as peripherally derived cells, occurs to varying degrees of severity and in a highly disease- and context-specific manner (DiSabato et al., 2016). While transient, acute inflammatory responses against CNS targeting pathogens such as bacteria and viruses can be life-saving, uncontrolled, dysregulated and misdirected neuroinflammatory responses against CNS pathogens and antigens can have devastating consequences such as bacterial/viral encephalitis and autoimmune diseases (DiSabato et al., 2016).

### Autoimmune Neuroinflammatory Disorders

Multiple sclerosis (MS), the most common neuroinflammatory disorder in adults, is a chronic autoimmune disease in which immune-mediated pathological CNS inflammation results in focal regions of demyelination and diffuse neurodegeneration (Lassmann, 2018). The inciting events that trigger the development of MS and the corresponding murine disease, experimental autoimmune encephalomyelitis (EAE), are incompletely understood. Two major theories, however, attempt to explain MS/EAE pathogenesis: (1) the CNS-extrinsic model, which proposes that peripherally activated autoreactive T-cells enter into circulation and subsequently gain access to the CNS by breaching one of the regulatory interfaces and (2) the CNS-intrinsic model, where the autoreactive T lymphocytes are hypothesized to infiltrate the CNS secondary to an inflammatory response initially triggered within the brain parenchyma (Dendrou et al., 2015). While these theories posit that T-cells act as the drivers of neuroinflammation in this context, recent studies demonstrating the efficacy of B-cell depleting therapies in limiting MS relapses have shifted attention toward further understanding MS as a disease of both the T- and B-cell compartment (Bar-Or et al., 2008; Hauser et al., 2008). Recent literature suggests that the chronic neuroinflammation that occurs in MS is the result of deregulated inflammatory responses occurring in both the periphery and within the CNS (Lassmann, 2003; Magliozzi et al., 2010; Michel et al., 2015). These findings thus, highlight the close integration of the central and peripheral immune systems, which may serve both distinct and parallel functions within the two compartments. Systemic immunologic dysfunction in patients with MS is characterized by impaired regulatory T-cell (T_Reg_) mediated suppression and the aberrant activation of peripheral immune cells, which subsequently infiltrate the CNS where they undergo reactivation (Kaskow and Baecher-Allan, 2018; Li et al., 2018).

The involvement of peripherally derived immune cells in MS/EAE pathogenesis is dependent on their ability to infiltrate the various compartments of the CNS, which can be accomplished by disrupting three routes: the BBB, the blood-meningeal barrier (BMB), and the blood-CSF barrier (BCSFB). It remains unknown, however, specifically which of these barriers is initially breached in MS/EAE. Disruption of the BBB is thought to be an early event in the development of MS/EAE, and episodes of recurrent BBB leakage occur over the course of the disease via multiple mechanisms (Argaw et al., 2012; Niu et al., 2019; Uchida et al., 2019; Zeitelhofer et al., 2020). Whether this impairment is an inciting event or secondary to another event that triggers pro-inflammatory cascade remains unknown. Recent evidence suggests that meningeal lymphoid aggregate formation may facilitate the production of inflammatory cytokines and chemokines which serve to activate CNS-resident astrocytes and microglia as well as facilitate the recruitment and activation of additional immune cells (Pikor et al., 2015). Given the evidence demonstrating microglial and astrocytic reactivity prior to appreciable immune infiltrate, it is possible that activation of these cells also provides the catalyst necessary to recruit peripheral immune cells into the brain parenchyma by way of compromised BBB and BMB integrity (D’Amelio et al., 1990; Wang et al., 2005; Pham et al., 2009; Zrzavy et al., 2017).

### Key Cellular Components

**FIGURE 3:**
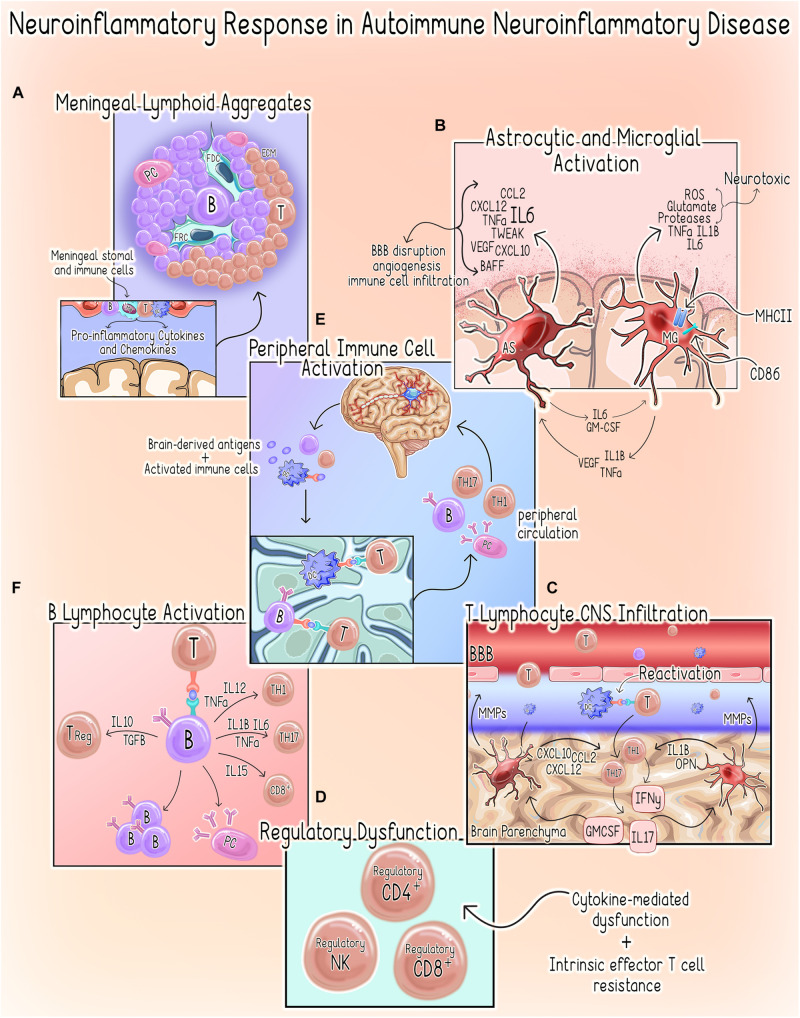
Neuroinflammatory response in autoimmune neuroinflammatory disease. **(A)** Meningeal Lymphoid Aggregates. Meningeal Lymphoid Aggregates resembling tertiary lymphoid tissues characterized by collections of lymphocytes, fibroblast reticular cells (FRC) and follicular dendritic cells (FDC) surrounded by an extracellular matrix (ECM) network occur in MS patients (Pikor et al., 2015). **(B)** Astrocyte (AS) and Microglial Activation (MG). Activation of microglia to a pro-inflammatory phenotype promotes increased expression of pro-inflammatory cytokines, MHCII and co-stimulatory molecule CD86, which primes them to act as antigen-presenting cells (Dong and Yong, 2019; Guerrero and Sicotte, 2020). Activated microglia can directly kill neurons by releasing neurotoxic factors such as reactive oxygen species (ROS), glutamate, proteases and the pro-inflammatory cytokines tumor necrosis factor alpha (TNFα), IL1β, IL6 (Dong and Yong, 2019; Guerrero and Sicotte, 2020). Microglial secretion of IL1, TNFα, and vascular endothelial growth factor beta (VEGF-B) stimulates astrocytes to adopt a pro-inflammatory/neurotoxic phenotype (Linnerbauer et al., 2020). Similarly, astrocytes can induce a neurotoxic phenotype in microglia via the secretion of IL6 and granulocyte-macrophage colony-stimulating factor (GM-CSF) (Linnerbauer et al., 2020). In the setting of autoimmune neuroinflammation, astrocytes are a primary source of IL6, which can have both protective or deleterious effects (Brambilla, 2019). Additionally, astrocytes also both produce and respond to TNF-related weak inducer of apoptosis (TWEAK). TWEAK-mediated stimulation of astrocytes induces NF-kβ signaling, which results in an increased secretion of pro-inflammatory cytokines and matrix metalloproteinases (MMPs) (Yepes, 2007; Stephan et al., 2013). Astrocytes also regulate immune cell recruitment through the expression of chemoattractant factors including chemokine ligand 2 (CCL2), stromal cell-derived factor-1 (CXCL12/SDF-1) and C-X-C motif chemokine ligand 10 (CXCL10) (Brambilla, 2019). The resultant effects include increased BBB permeability, angiogenesis and initiation of a neuroinflammatory cascade through the promotion of immune cell infiltration (Lynch et al., 1999; Yepes, 2007; Stephan et al., 2013). **(C)** T Lymphocyte CNS Infiltration. Both astrocytes and microglia produce MMPs, which contribute to BBB permeability allowing for increased infiltration of peripheral immune cells (Abbott et al., 2006; Zia et al., 2020). Within the CNS, dendritic cells (DCs) promote T-cell antigen reactivation allowing them to infiltrate the parenchyma where they can activate astrocytes and microglia to assume pro-inflammatory phenotypes through secretion of IFNγ, IL-17 and GM-CSF (Dong and Yong, 2019; Guerrero and Sicotte, 2020). Likewise, microglia-derived factors such as IL-1β and osteopontin (OPN) promote CD4+ T-cell expansion and polarization of T_h_1 and T_h_17 responses, respectively (Schetters et al., 2017; Dong and Yong, 2019). **(D)** Regulatory Dysfunction. Several cytokines present in the neuroinflammatory microenvironment (e.g., IL-6, IL-7, IL-18, IL-15, and TNF-α) are known to compromise T_Reg_ function (Walker, 2009). There is also evidence to suggest that an intrinsic resistance of effector T-cells may also contribute to this phenomenon in MS (Walker, 2009). CD4^+^ T-cells isolated from MS patients demonstrate resistance to CD4+,CD8^+^ and NK T_Regs_ (Correale and Villa, 2008; Baughman et al., 2011; Nielsen et al., 2012; Gross et al., 2016; Kaskow and Baecher-Allan, 2018). **(E)** Peripheral Immune Cell Activation. Following initial injury, persistent neuronal damage results in the release of self-antigens and egress of activated immune cells to the cervical lymph nodes by way of the meningeal lymphatics system (McMahon et al., 2005; Quintana et al., 2014). DCs are shown to migrate from the brain parenchyma to the deep cervical lymph nodes where they can prime autoreactive T-cells (orange) (Clarkson et al., 2017). Additionally, B-cells (purple) sharing clonality with those populating the CNS have been demonstrated in the draining cervical lymph nodes of MS patients, suggesting a role for peripheral B- and plasma cell (PC) expansion in disease pathogenesis (Palanichamy et al., 2014; Stern et al., 2014). These autoreactive T-cells, B-cells and plasma cells enter systemic circulation and can infiltrate the CNS where they contribute to the perpetuation of the neuroinflammatory response. **(F)** B Lymphocyte Activation. B-cells can secrete both pro- and anti-inflammatory cytokines, which support a dual role for these cells in promoting and limiting inflammatory responses (Bar-Or et al., 2010; Shen and Fillatreau, 2015; Li et al., 2018). In the periphery, B-cells can also drive aberrant activation of autoreactive T_h_1, T_h_17, and CD8^+^ T-cells that will subsequently infiltrate the CNS through the secretion of Pierce et al. (1988) and Rivera et al. (2001).

Within the CNS, the neuroinflammatory response occurs in a compartmentalized fashion with chronically activated microglia and other immune cell (summarized in Table 1) aggregates within the meninges and brain parenchyma contributing uniquely to MS/EAE pathogenesis (Magliozzi et al., 2007, 2010; Zrzavy et al., 2017; Figure 3).

**TABLE 1:** Contribution of distinct cell types to autoimmune neuroinflammation.

Cell type	Cellular subsets	Cell profile/differential gene expression	Factors produced	Contribution to disease pathogenesis	References
Astrocyte**s**	Heterogeneous population with phenotypic spectrum ranging from pro- to anti-inflammatory	*Gfap*, *C3*, *C4b, H2-Aa, Cd274*, *Mafg, Nrf2* Downregulation of homeostatic markers (e.g., *Cnx43, Btbd17, Apoe, Aldh1l1, Slc1a2, Slc1a3, Aqp4*, and *Fgfr3*)	TGF-β IL-4, IL-6 IL-10, GM-CSF CCL2 CXCL12/SDF1 CXCL10 BAFF TWEAK	Phagocytosis and glial scar formation Regulation of microglial phenotype Recruitment of peripheral immune cells Promote MMP9-mediated BBB disruption Support B cell function and survival	Itoh et al., 2018; Brambilla, 2019; Wheeler et al., 2020; Linnerbauer et al., 2020; Borggrewe et al., 2021
Microglia	Heterogeneous population with phenotypic spectrum ranging from pro- (M1) to anti-inflammatory (M2)	*CTSD, APOC1, GPNMB, ANXA2, LGALS1, CD74, LYZ, CX3CR1, SLC2A5, SPP1, PADI2, LPL HLA- (-DRA, -DRB1, -DBP1) Ly86, Ccl2, Cxcl10, Mki67* Downregulation of homeostatic markers *P2ry12, Tmem119*, and *Selplg*	ROS Glutamate Proteases TNF-a Interleukins: IL-1, IL-4, IL-6, IL-10, IL-13, IL-33 TGF-β Osteopontin CXCL10 CCL2 CCL5	Neuronal oxidative injury Phagocytosis and antigen presentation Recruitment of peripheral immune cells Promote T cell expansion and polarization to TH1 and TH17 cells Regulation of astrocytic phenotype	Schetters et al., 2017; Dong and Yong, 2019; Jordão et al., 2019; Masuda et al., 2019; Guerrero and Sicotte, 2020; Masuda et al., 2020; Acharjee et al., 2021
Bone marrow-derived monocytes	Heterogeneous population with phenotypic spectrum ranging from pro- (M1) to anti-inflammatory (M2)	*Cd44, Cd49e, Cd74 Lag3, Nuak1, Olfml3, Rtn1, Sall3, Slc1a3, Ly6c2, Ccr2, Fn1*	IL-1β	Promote IL-17 production from T cells Antigen presentation Differentiation into macrophages and DCs	Ajami et al., 2011; Ajami et al., 2018; Jordão et al., 2019; Monaghan et al., 2019; Grassivaro et al., 2020; McGinley et al., 2020
T cells	TH1	*CCL3, CCL4, CCL5 GZMB, ICOS, IL3, IL7R LAG3, STAT4, TBX21*	IL-6 IL-17 IFN-γ GM-CSF	Promote microglial activation, TNF-a production, and expression of MHCII, CD80 and CD86 Activation of pro-inflammatory astrocytes Enhance antigen presenting capacity of cells in the meningeal and perivascular spaces	Correale and Villa, 2008; Absinta et al., 2015; Hu et al., 2017; Kaskow and Baecher-Allan, 2018; Mundt et al., 2019
	TH17	*IL22, CCL3, CCL4, CCL5 GZMB, ICOS, IL3, IL7R LAG3, STAT4, TBX21*	IL-17 IL-21 IFN-γ GM-CSF	Activation of microglia and astrocytes Recruitment of BMDMs, neutrophils and lymphocytes Disruption of BBB	Hu et al., 2017; McGinley et al., 2020
	CD8	GZMB NFAT2 CD103	IFN-γ TNF-α IL-17 GM-CSF	Neuronal damage Disruption of BBB	Machado-Santos et al., 2018; Stojić-Vukanić et al., 2020
Dendritic cells (DCs)	cDC	CD11c, MHC II *Flt3, Zbtb46, Batf3, Itgae, Clec9aå*	IL-1β IL-6 IL-13 TGF-β	Promote differentiation of effector T_*H*_1 and T_*H*_17 cells Antigen reconfirmation of autoreactive T cells Promote CD8+ T cell response Activation of autoreactive T cells in deep cervical lymph nodes Promote T cell pro-inflammatory cytokine and chemokines secretion	Dittel et al., 1999; Bailey et al., 2006, 2007; Stasiolek et al., 2006; Diebold, 2008; Monaghan et al., 2019; Bossù et al., 2015
	pDC	CD4, CD68, CD123 HLA-DR ILT-3	IL-6 IL-10 Impaired IFN-α	Suppression of cDC-dependent induction of T_*H*_17 and T_*H*_1 responses IDO-independent suppression of T cell cytokine production	Stasiolek et al., 2006; Bailey-Bucktrout et al., 2008; Mitchell et al., 2018
**B cell**	Effector and class-switched memory B-cells	CD20, CD19 CD27, CD38, CD70 CXCR4	IL-6 TNF-α GM-CSF	Antigen presentation Activation of autoreactive T-cells Immunoglobulin synthesis Differentiation of plasmablasts and plasma cells	Pierce et al., 1988; Rivera et al., 2001; Cepok et al., 2006; Machado-Santos et al., 2018; Häusser-Kinzel and Weber, 2019; Ramesh et al., 2020

### Astrocytes

Astrocyte reactivity begins during the earliest stages of MS lesion formation and persists even after immune cells retreat (Brambilla, 2019). In MS/EAE signs of astrocyte activation are noted even before CNS immune infiltration (D’Amelio et al., 1990; Wang et al., 2005; Pham et al., 2009). Astrocytes localized to the leading edges of active lesions exhibit intracellular lipid inclusions, reflective of their role in the phagocytosis of myelin debris, whereas those adjacent to immune cell infiltrates demonstrate increased reactivity (Ponath et al., 2017, 2018). In chronic lesions, astrocytes demonstrate a persistent, but diminished reactivity and concentrate at the lesion rim (Brosnan and Raine, 2013; Brambilla, 2019). As chronic lesions become inactive, the astrocytes acquire GFAP+ filaments suggesting astroglial scar formation, which serves as an attempt to limit neurotoxic inflammation (Brambilla et al., 2009; Brosnan and Raine, 2013; Ludwin et al., 2016; Ponath et al., 2018). Beyond the lesions, astrocytes of the adjacent gray and white matter also demonstrate persistent signs of subtle activation (Brambilla, 2019). Astrocytes play a neuroprotective role early in disease by restricting the infiltration of immune cells, likely through maintenance of BBB integrity and FasL-mediated T-cell apoptosis (Choi et al., 1999; Voskuhl et al., 2009; Wang et al., 2013; Mayo et al., 2014) and conversely, astrocyte depletion during the chronic phase of disease produces improved outcomes and decreased CNS leukocyte infiltration, suggesting a deleterious role later in disease pathogenesis (Mayo et al., 2014). A more recent study utilizing FACs-isolation of astrocytes from distinct anatomical regions in the setting of EAE, supports the above findings by providing additional evidence that, during the neuroinflammatory response, astrocytes exist as a heterogeneous population characterized by unique transcriptional profiles suggestive of distinct functions (Borggrewe et al., 2021). Furthermore, Borggrewe et al. (2021) found that the unique transcriptional response of astrocyte subsets exhibited additional variation depending on the stage of disease and anatomical location (e.g., acute vs. chronic). Astrocytes isolated from the spinal cord of mice with EAE demonstrated decreased expression of genes associated with a homeostatic signature including, those involved in maintenance of the BBB, cholesterol synthesis and neuronal support (Itoh et al., 2018; Borggrewe et al., 2021). Not surprisingly, genes involved in neuroinflammation and neurotoxicity were noted to be increased (Itoh et al., 2018; Wheeler et al., 2020; Borggrewe et al., 2021). Conversely, during chronic stages gene expression signatures were consistent with a proliferative profile, which may reflect the transition to a regenerative/protective phenotype (Borggrewe et al., 2021).

### Microglia

In active MS/EAE lesions, microglia with a pro-inflammatory/neurotoxic (M1) phenotype predominate in the early stages of demyelination (Zrzavy et al., 2017). These microglia express molecules involved in phagocytosis, antigen presentation, T-cell co-stimulation, and oxidative injury (Zrzavy et al., 2017; Böttcher et al., 2020). Additionally, they exhibit decreased expression of the homeostatic genes P2RY12, TMEM119, CX3CR1, and GPR56 (Zrzavy et al., 2017; Böttcher et al., 2020). Recent data suggests that IgG immune complexes (IgG-IC) bound to myelin may activate microglia by decreasing toll-like receptor (TLR) tolerance in an FcyRI and FCyRIIa-dependent manner (van der Poel et al., 2020). When stimulated with both the TLR3 agonist Poly I:C and IgG-IC these microglia demonstrate increased production of pro-inflammatory chemokines and cytokines (van der Poel et al., 2020). As the lesion progresses, peripheral macrophages are increasingly recruited and, along with resident microglia, adopt an intermediate phenotype between neurotoxic (M1) and neuroprotective (M2) (Zrzavy et al., 2017). Progression of the lesion to a slowly expanding or chronic lesion, is characterized by a decrease in the total density of microglia/macrophages with the remaining phagocytes localizing to the lesion rim where they are noted to contain products of myelin degradation and characterized predominantly by an M1-phenotype (Zrzavy et al., 2017; Jäckle et al., 2020). The density of macrophage- and microglia-like cells significantly decreases in completely inactive lesions and microglial P2RY12 expression reappears. Interestingly, despite P2RY12 expression, a majority of these cells also express markers associated with a neurotoxic (M1) activation phenotype (Zrzavy et al., 2017). These temporal and regional changes are reflective of the specific cellular functions. Early in disease, a neurotoxic (M1) phenotype predominates, reflecting microglial response to an acute disruption of CNS homeostasis (Zrzavy et al., 2017). Myelin phagocytosis promotes polarization to a neuroprotective (M2) phenotype, with the maximum expression of M2-like markers detected in the lesion core where re-myelination begins (Zrzavy et al., 2017). These microglia produce cytokines involved in the resolution of inflammation and tissue repair including interleukins (IL-) 4, 10, 13, and 33, as well as transforming growth factor beta (TGF-β) (Chu et al., 2018). Conversely, cells at the periphery possess a phagocytic phenotype (Zrzavy et al., 2017).

Interestingly, microglial reactivity is not restricted to MS lesions, as the normal appearing white matter (NAWM) of MS patients is characterized by a reduced number of microglia expressing P2RY12 and a moderate increase in pro-inflammatory markers (Zrzavy et al., 2017). While this may be reflective of the diffuse effects of a chronic inflammatory environment, it may also indicate adjacent leptomeningeal inflammation, which has shown to occur in some patients with MS and to be associated with adjacent cortical demyelination (Absinta et al., 2015). However, despite exhibiting an activated phenotype, studies have found that microglia within the NAWM of MS brains exhibit a decreased inflammatory responsiveness (Guerrero and Sicotte, 2020). This finding was true of both acute and chronic disease, suggesting that the chronic inflammatory environment may induce a persistent hypo-responsive activated state in microglia of the NAWM, distant from focal lesions (Zrzavy et al., 2017; Guerrero and Sicotte, 2020).

Elucidating the specific contributions of microglia to MS/EAE pathogenesis has been complicated by the inability to distinguish CNS-infiltrating bone marrow-derived monocytes (BMDM) from CNS-resident microglia (Böttcher et al., 2020). Recent technological advances permitting analysis at the single-cell level (reviewed in Papalexi and Satija, 2018) have not only allowed for this distinction, but have also provided greater insight into the mechanisms underlying the vast cellular plasticity and heterogeneity of CNS-resident microglia (Masuda et al., 2019; Böttcher et al., 2020). These studies, which are reviewed extensively in Miedema et al. (2020), provide additional evidence that discrete microglial subsets perform multiple, unique functions and contribute to MS/EAE disease pathogenesis through the temporal regulation of disease- and context-specific gene expression programs (Masuda et al., 2019; Böttcher et al., 2020).

### Bone Marrow-Derived Monocytes

Bone marrow-derived monocytes are recruited to the inflamed CNS and, upon entry, assume a range of phenotypes that share extensive similarities with CNS-resident microglia (Plemel et al., 2020). While studies in EAE suggest that microglial activation is important for initiation of EAE, disease progression is reported to depend largely on BMDMs (Brosnan et al., 1981; Huitinga et al., 1990; Bauer et al., 1995; Ponomarev et al., 2005). These studies are in agreement with human data, which suggests that early disease is characterized by significant microglia activation with little to no bone marrow-derived phagocytes present in newly forming lesions (Barnett and Prineas, 2004). The concept of temporally distinct roles for microglia and BMDMs in disease pathogenesis is further supported by work of Ajami et al. (2011) which demonstrates that increased infiltration of BMDMs corresponds with a marked decrease in CNS-resident microglia in mice during more severe stages of EAE. Additionally, in the early stages of EAE, increased infiltration of BMDMs has been suggested to correlate with increased disease severity (Ajami et al., 2011; Lewis et al., 2014). Interestingly, Plemel et al. (2020) recently found that, while BMDMs acutely infiltrate the CNS in response to demyelinating injury, microglia demonstrate a progressive predominance within the lesions and their ablation results in the increased accumulation of BMDMs. The results of this study suggest that microglia may play a protective role by limiting the infiltration and function of BMDM through regulation of their recruitment, entry, proliferation and survival (Ajami et al., 2011; Plemel et al., 2020).

As with microglia, elucidating the specific contributions of BMDMs to MS/EAE pathogenesis has been complicated by their existence as a heterogeneous, highly plastic population that has proven difficult to distinguish from other cellular compartments (Plemel et al., 2020). Recent evidence suggests the possibility that previous studies may have misidentified CNS-resident microglia as BMDMs (Plemel et al., 2020). There is also evidence to suggest that early in development and during states of chronic neuroinflammation, CNS-infiltrating BMDMs may acquire expression of markers conventionally thought to be restricted to microglia (Caravagna et al., 2018; Grassivaro et al., 2020). Additionally, it was historically believed that BMDMs served as precursors to terminally differentiated macrophages (Mφ) and dendritic cells (DCs) (Guilliams et al., 2018). Recent advances in our understanding of monopoiesis, suggest that circulating monocytes possess functional properties prior to infiltrating the tissues and that monocyte-derived cells recruited to areas of inflammation represent a population of cells that is distinct from both DCs and tissue resident Mφs (Guilliams et al., 2018). Thus, studies in which peripherally derived monocytes and macrophages were evaluated as a single entity may suggest the presence of heterogeneous subsets rather than distinct cellular compartments.

Advances in both technology and understanding have begun to shed light on the contribution of BMDMs to the neuroinflammatory response. *In vivo* imaging studies suggest that BMDMs, like microglia and astrocytes, adapt their phenotype in a temporally and regionally dependent manner (Locatelli et al., 2018). Furthermore, through the use of massively parallel single-cell RNA-sequencing (MARS-seq) Giladi et al. (2020) identified eight distinct monocyte subsets infiltrating the inflamed CNS of mice with EAE in a disease stage-dependent manner and suggest that pathogenicity may be specific to certain monocyte subpopulations (e.g., *Cxcl10*+). This premise that specific BMDM subsets may assume divergent roles is in agreement with the finding that BMDMs may also adopt suppressive, immunoregulatory phenotypes (Zhu et al., 2007; Giladi et al., 2020). The results of these studies, in combination with the varied effects of BMDM depletion at different stages of EAE, further underscore the importance of understanding the context-specific contributions of this heterogeneous cellular compartment (Brosnan et al., 1981; Huitinga et al., 1990; Moreno et al., 2016).

### T Lymphocytes

In steady-state, T-cells are essentially absent from the brain parenchyma and, thus their contribution to the maintenance of CNS homeostasis has remained relatively unexplored (Prinz and Priller, 2017). In MS, T cells are detected early within parenchymal lesions and autoreactive CD4^+^ T-cells are also present in the circulation and lymph nodes of MS patients (Bielekova et al., 2004; Prinz and Priller, 2017). Several distinct effector T-cell subsets have been implicated in the pathogenesis of MS/EAE, and details of their involvement are outlined in Table 1. Importantly, in human MS patients, MHC class I restricted CD8+ T cells predominate in lesions of all stages of human disease, whereas CD4+ T cells predominate in EAE (Machado-Santos et al., 2018). T cell infiltration is significantly higher in early stages of MS compared with progressive disease and also in active verses inactive lesions (Machado-Santos et al., 2018). Furthermore, features associated with tissue-resident memory cells, including CD8α/α double positivity, are noted to characterize a majority of CD8+ T cells in human MS lesions (Machado-Santos et al., 2018).

In addition to the autoreactivity of the CD4+ and CD8+ T-cells, effector T-cells isolated from MS patients demonstrate resistance to the suppressive functions of regulatory T-cells (Kaskow and Baecher-Allan, 2018). This is thought to occur as a result of both an intrinsic resistance of effector T-cells as well as suppression of regulatory T cell function by cytokines within the neuroinflammatory milleu (e.g., IL-6, IL-7, IL-18, IL-15, and TNF-α) (Walker, 2009). A recent study in EAE, found that Piezo1, a mechanosensitive ion channel known for its role in the inflammatory response to bacterial pathogens and cancer, may contribute to MS/EAE pathogenesis through selective repression of regulatory T cells (Jairaman et al., 2021). Accordingly, deletion of Piezo1 in regulatory T cells significantly attenuated EAE disease severity (Jairaman et al., 2021).

### Dendritic Cells (DCs)

Dendritic cells, the professional antigen presenting cells, are thought to contribute to both the resolution and pathogenesis of autoimmune neuroinflammatory disease through functions in the periphery and within the CNS (Dittel et al., 1999; Bossù et al., 2015; Mitchell et al., 2018). In the periphery, activation of autoreactive CD4^+^ T-cells by classical DCs (cDCs) promotes their differentiation into effector T_h_1 and T_h_17 cells, which can subsequently infiltrate the CNS (Diebold, 2008). In contrast, plasmacytoid DCs (pDCs) appear to primarily limit neuroinflammation in the setting of MS/EAE. Specifically, pDCs have been shown to suppress cDC-dependent induction of T_h_17 and T_h_1 responses (Bailey et al., 2006, 2007; Stasiolek et al., 2006; Bailey-Bucktrout et al., 2008). Furthermore, pDCs isolated from the peripheral blood of patients with MS exhibited an immature phenotype characterized by impaired stimulatory capacity and IFN-α production, suggesting an immunosuppressive phenotype (Bailey et al., 2006, 2007; Stasiolek et al., 2006; Bailey-Bucktrout et al., 2008).

While the process of CNS antigen presentation remains poorly understood, recent evidence suggests that cDCs may contribute to autoimmune neuroinflammatory disorder pathogenesis by migrating from the brain parenchyma to the deep cervical lymph nodes where they prime autoreactive T-cells in a CCR7-dependent manner (Clarkson et al., 2017). Although the presence of resident DCs in the human CNS parenchyma at steady-state has not been proven conclusively, our group previously identified resident cDCs and pDCs in equal proportion in the brain parenchyma of mice at steady state (Dey et al., 2015). In response to disruptions of CNS homeostasis, cDCs and pDCs readily accumulate in the choroid plexus, leptomeninges, and perivascular space (McMenamin, 1999; Pashenkov et al., 2001; Bailey et al., 2007; Mitchell et al., 2018; Jordão et al., 2019). In the context of MS, in addition to the CSF, cDCs infiltrate white matter lesions and the leptomeninges (Serafini et al., 2006; Lande et al., 2008). The trafficking of DCs into the CNS is thought to primarily promote T-cell antigen reconfirmation upon CNS entry, which induces their production of pro-inflammatory cytokines (e.g., IL-2, IFNγ, and TNFα) and chemokines (e.g., CCL3 and CCL5) (Bailey et al., 2006, 2007; Goverman, 2009; Prodinger et al., 2011). A recent study utilizing single-cell analysis also found that CNS-cDCs demonstrated increased expression of MHCII compared to peripheral blood cDCs in patients with MS, suggesting that these CNS-cDCs are functionally primed for antigen-presentation and activation of autoreactive T-cells (Esaulova et al., 2020). In addition to their role as antigen-presenting cells, cDCs promote CD8^+^ T-cell responses and the differentiation of T_h_17 through the secretion of pro-inflammatory cytokines including IL-1β, IL-6, IL-13, and TGF-β (Codarri et al., 2011; El-Behi et al., 2011; Luessi et al., 2016; Malo et al., 2018).

### B Lymphocytes

In addition to immunoglobulin synthesis, B-cells can also function as antigen presenting cells, and they are especially critical for the priming of T-cells in the periphery when antigen concentrations are low (Pierce et al., 1988; Rivera et al., 2001). The success of B-cell depleting therapies in limiting relapses and the identification of B-cell-rich immune cell aggregates in the meninges, parenchyma and/or CSF in patients with MS has drawn attention to the potential role of B-cells as contributors to MS neuroinflammation in both the periphery and CNS (Magliozzi et al., 2007). The B-cells found within the CNS are largely class-switched memory B-cells and demonstrate increased clonal expansion in the CSF compared to the blood suggesting that an antigen-driven selection process drives the accumulation of B-cells in CNS compartments (Cepok et al., 2006). In the periphery, B-cells can drive aberrant activation of autoreactive T-cells that will subsequently infiltrate the CNS. Additionally, B-cells sharing clonality with those populating the CNS have been demonstrated in the draining cervical lymph nodes of MS patients (Palanichamy et al., 2014; Stern et al., 2014). This is of particular interest as it could provide a mechanism for the “epitope spread” seen in some patients with MS (McMahon et al., 2005; Quintana et al., 2014). Epitope spread refers to a phenomenon in which additional epitopes are released secondary to CNS damage resulting in a shift in the attack toward different antigenic targets and may explain the diffuse cortical damage and progressive nature some types of MS (McMahon et al., 2005; Quintana et al., 2014).

The above studies highlight the significant complexity of the neuroinflammatory response, which arises as a consequence of the vast heterogeneity and plasticity of the individual components comprising the neuro-immune axis. The contribution of the above cell types to MS/EAE pathogenesis is characterized by both marked temporal and regional heterogeneity, allowing for the acquisition of both protective and deleterious phenotypes in a highly context specific manner (Plastini et al., 2020). Accordingly, studies have demonstrated that the broad depletion or manipulation of specific cellular subsets can have both therapeutic and detrimental effects depending on a range of factors including, disease stage (Brosnan et al., 1981; Huitinga et al., 1990; Moreno et al., 2016). These and the above findings emphasize that the development of effective therapeutic strategies relying on the manipulation of components of the neuro-immune axis will greatly depend on understanding the development, recruitment, regulation, interactions and functions of above cellular components during specific stages of neuroinflammatory disease.

## Primary Brain Tumor and Exploitation of Neuroinflammation

The contributions of the individual components of the neuro-immune axis to the neuroinflammatory response exhibit significant disease-specificity. However, despite these disease-specific differences, the fundamental characteristics of significant cellular heterogeneity, cellular plasticity, mechanistic non-redundancy (multiple, distinct mechanisms capable of producing same outcome) and functional redundancy (single mechanisms capable of producing multiple, distinct outcomes) are shared across the range of pathologies. Accordingly, when co-opted by tumors, the neuroinflammatory response can directly promote tumor progression and survival (Mantovani et al., 2008). Throughout the stages of tumor development, host inflammatory responses make distinct contributions through both pro- and anti-tumorigenic effects (Greten and Grivennikov, 2019). These complex interactions between the components of the host inflammatory response, surrounding stroma, and developing tumor create a survival niche known as the tumor microenvironment (TME) (Greten and Grivennikov, 2019).

### Key Cellular Components

**FIGURE 4:**
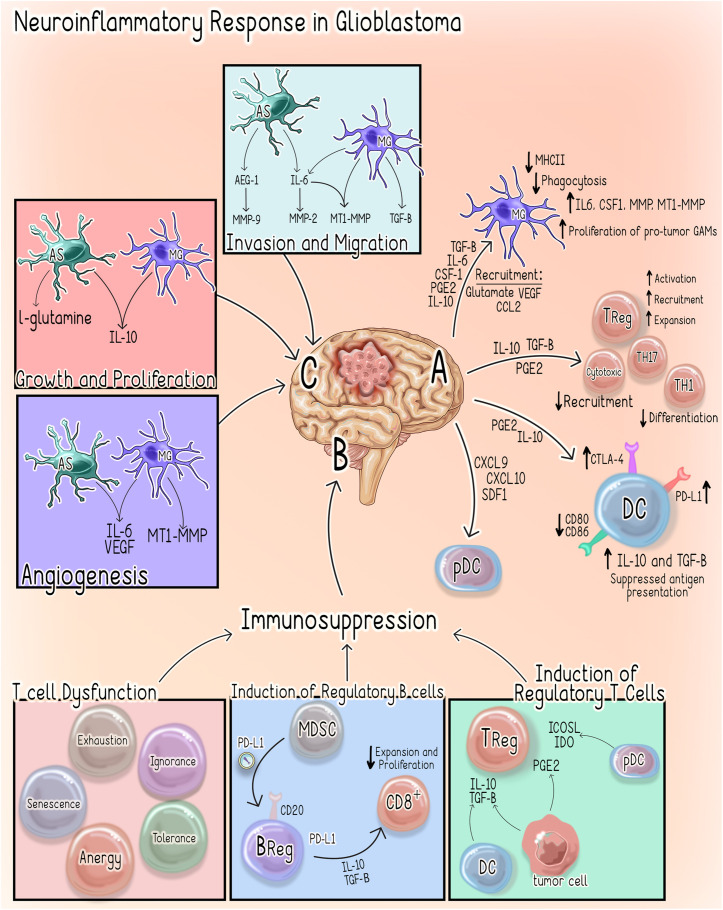
Neuroinflammatory response in Glioblastoma. **(A)** Glioma-Derived Factors. Glioma cells recruit microglia/macrophages to the TME by secreting chemoattractant factors including, monocyte chemoattractant protein-1 (MCP-1/CCL2), vascular endothelial growth factor (VEGF) and glutamate (Li and Graeber, 2012). Several glioma-derived factors including TGF-β (Suzumura et al., 1993), prostaglandin E2 (PGE2), macrophage colony-stimulating factor (M-CSF/CSF-1) and interleukins 6, and 10 promote TAMs to assume pro-tumor phenotypes characterized by decreased expression of MHCII, decreased phagocytic capacity and increased production of IL-6, CSF-1, matrix metallopeptidases (MMPs) and microglial membrane-type 1 matrix metalloproteinase (MTI-MMPs) (Suzumura et al., 1993; Taniguchi et al., 2000; Mitrasinovic et al., 2001; Mantovani et al., 2002; Nakano et al., 2008; Held-Feindt et al., 2010; Li and Graeber, 2012; Pyonteck et al., 2013). Furthermore, glioma cells produce factors (e.g., M-CSF/CSF-1), which subsequently drive the proliferation of these pro-tumor microglia (Forstreuter et al., 2002). IL-10, PGE2, and TGF-β also suppress the function of effector T-cell subsets, while simultaneously inducing regulatory T-cell activation and expansion (Ooi et al., 2014). PGE2 and IL-10 suppress DC antigen presenting function by inducing increased expression of immune checkpoint molecules (PD-L1 and CTLA4),downregulating co-stimulatory molecules CD80/86 and increasing production of IL-10 and TGF-B (De Smedt et al., 1997; Hilkens et al., 1997; Kaliński et al., 1998). GBM cells recruit immunosuppressive pDCs to the TME through the secretion of CXCL9, CXCL10 and CXCL12/SDF-1 (Mitchell et al., 2018). **(B)** Immunosuppression. GBM-mediated T-cell dysfunction as well as regulatory B- and T-cell induction contribute to the immunosuppressive microenvironment that facilitates GBM tumorigenesis (Mitchell et al., 2018; Woroniecka et al., 2018; Lee-Chang et al., 2019) **(C)** Microglia and Astrocytes. Microglia and Astrocytes can promote the invasion and migration of GBM through the secretion of AEG-1 and TGF-B, respectively (Taniguchi et al., 2000; Badie and Schartner, 2001; Gabrusiewicz et al., 2011; Coniglio et al., 2012; Li and Graeber, 2012; Hambardzumyan et al., 2016; Guan et al., 2018; DeCordova et al., 2020). IL-6 is also secreted by both astrocytes and microglia is shown to promote GBM invasion through increased expression of MMP-2, MT1-MMP/MMP-14 (Li and Graeber, 2012; Guan et al., 2018). Similarly, astrocyte elevated gene-1 (AEG-1) upregulates NF-kβ-mediated expression of MMP-2 and MMP-9 which promote the proliferation and invasion (Guan et al., 2018). GBM is also dependent on astrocytes to supply nutrients, such as L-glutamine, which is necessary for its survival and proliferation (DeBerardinis et al., 2007; Tardito et al., 2015). Both astrocytes and microglia secrete IL-10, which also facilitates GBM growth and proliferation (Li and Graeber, 2012). Lastly, GAAs and MGs directly promote angiogenesis through the secretion of IL-6, VEGF and MT1-MMP (Li and Graeber, 2012; Guan et al., 2018). AS, Astrocyte; MG, Microglia; DC, Dendritic Cell; pDC, plasmacytoid dendritic cell; MDSC, myeloid derived suppressor cell; TH1, T helper 1; TH17- T helper 17; Treg, Regulatory T cell; BReg, Regulatory B cell.

Glioblastoma (GBM), the most common primary brain tumor in adults, is one of the deadliest cancers and it develops in relatively “immune privileged” CNS environment behind the BBB where it is known to generate a highly immunosuppressive TME (DeCordova et al., 2020). Unlike autoimmune neuroinflammatory disease in which uncontrolled hyperactivity of the immune system results in neurotoxicity, in the context of GBM, the neoplastic cells induce structural and functional changes or damage to the surrounding CNS tissue by actively influencing and manipulating the neuro-immune axis towards a suppressive phenotype. In order to carry out such an elegant task, glioma cells secrete a variety of cytokines, chemokines, and growth factors, which allows them to recruit and hijack cells both within the CNS and the periphery (Table 2). These non-neoplastic cells work in concert to create a unique immunosuppressive microenvironment that promotes tumor growth, survival, and invasion (Hattermann et al., 2014; Broekman et al., 2018; Brandao et al., 2019; DeCordova et al., 2020; Mohme and Neidert, 2020; Wang et al., 2021) (Figure 4; summarized in Table 3).

**TABLE 2:** Effect of glioma-derived factors on non-neoplastic cells of GME.

Glioma-derived factors	Effects on non-neoplastic cells of GME	References
TGF-β	TAMs: Promotes pro-tumor (M2) phenotype, Downregulates expression of MHC I and II Suppresses phagocytosis T cells: Promotes activation and expansion of TRegs Suppression of cytotoxic T cells DCs: Impairs pDC function	Oh and Li, 2013; Ooi et al., 2014; Gieryng et al., 2017; Mitchell et al., 2018; DeCordova et al., 2020
IL-10	T cells: Suppresses recruitment of cytotoxic T cells Promotes activation and expansion of TRegs Prevents cDC-mediated development of TH1 cells via impaired IL-12 secretion DCs: Promotes cDCs to adopt immunosuppressive phenotypes Promotes secretion of IL-10 and TGF-β Downregulates expression of MHC and co-stimulatory molecules	De Smedt et al., 1997; Hilkens et al., 1997; Kaliński et al., 1998; Ooi et al., 2014; Mannino et al., 2015; Zong et al., 2016; DeCordova et al., 2020
Prostaglandin E2	T cells: Promotes development and expansion of TRegs Prevents cDC-mediated development of TH1 cells via impaired IL-12 secretion B cells: Promotes development of regulatory B cells DCs: Promotes cDCs acquisition of immunosuppressive phenotypes Promotes secretion of IL-10 and TGF-β	De Smedt et al., 1997; Hilkens et al., 1997; Kaliński et al., 1998; Miller et al., 2007; Ooi et al., 2014; Mannino et al., 2015; Zong et al., 2016
CSF-1	TAMs: Promotes proliferation and infiltration Promotes expression of IL-6 and CSF-1 DCs: Promotes cDCs acquisition of immunosuppressive phenotype Promotes secretion of IL-10 and TGF-β	Forstreuter et al., 2002; Zong et al., 2016; Gieryng et al., 2017
CCL2/MCP1	Promotes recruitment of TAMs, macrophages, monocytes, NK and T cells	Gieryng et al., 2017
VEGF	TAMs: Promotes recruitment and proliferation DCs: Promotes cDCs acquisition of immunosuppressive phenotype Promotes secretion of IL-10 and TGF-β	Forstreuter et al., 2002; Zong et al., 2016
Glutamate	Recruitment of TAMs	Li and Graeber, 2012
CX3CL1	Enhances recruitment and MMP expression in TAMs	Li and Graeber, 2012
CXCL9, CXCL10, CXCL12/SDF-1	Recruitment of pDCs	Mitchell et al., 2018

**TABLE 3:** Contribution of distinct cell types to glioblastoma-associated neuroinflammation.

Cell type	Cellular subsets	Cell profile/differential gene expression	Factors produced	Contribution to disease pathogenesis	References
Astrocytes	Heterogeneous population with phenotypic spectrum ranging from pro-to anti-tumor	*CHI3L1, HLA-DRA, HLA-DRB3, CD274, CD37, MKI67, ANXA2, C1S, C1R Downregulation of IFNγ and IFNα regulated genes (e.g., IFI44L, ISG15, IFIT3, IFI6)*	IL-6 IL-10 TGF-β AEG-1 L-glutamine CCL20	Promote tumor invasion, migration and proliferation Inhibition of antigen presentation and inflammatory cytokine production Suppression of T cell antigen-specific responses Induction of pDC dysfunction	Tardito et al., 2015; Guan et al., 2018; Jin et al., 2018; Henrik Heiland et al., 2019
Tumor associated-microglia/macrophages (TAMs)	Heterogeneous population with phenotypic spectrum ranging from pro-to anti-tumor	*HIF1A, VEGFA IFI44, SPP1 HLA-DRA, APOE CD163* Downregulation of microglial core genes (e.g., *CX3CR1, SELPLG*)	IL-6 IL-10 TGF-β MT1-MMP	Promote tumor invasion, migration, proliferation and angiogenesis Inhibition of antigen presentation and inflammatory cytokine production Suppression of T cell antigen-specific responses Induction of pDC dysfunction	Masuda et al., 2019, Masuda et al., 2020; Sankowski et al., 2019
T cells	CD4+ Regulatory T Cells	FOXP3 *Decorin*	IL-10 TGF-β	Suppression of effector T production of cytokines Suppression of effector T cell antigen-specific response	Fecci et al., 2006; Pallandre et al., 2007; Ooi et al., 2014; Romani et al., 2018; Huff et al., 2019; Bam et al., 2021; Leko et al., 2021; Mathewson et al., 2021
	CD4+ T helper cells	PRF1, GZMA, GZMH HAVCR2 (Th1) RORC (Th17) PD-1, CTLA-4, TIM-3, LAG-3, BTLA, TIGIT, CD39 Downregulation of CD28			
	CD8+ T cells	GZMB, NKG7, KLRB1, KLRD1			
Dendritic cells	cDC	PD-L1, CTLA-4 Downregulation of MHC and CD80/86	IL-10 IL-12 TGF-β	Suppression of cytotoxic T cell recruitment and TH1 cell differentiation Promote T cell anergy and apoptosis Promotes activation of TRegs	De Smedt et al., 1997; Hilkens et al., 1997; Kaliński et al., 1998; Dey et al., 2015; Zong et al., 2016; Mitchell et al., 2018; Patente et al., 2018
	pDC	CD4, CD68, CD123 HLA-DR ILT-3	Impaired IFNα	PD-L1/PD-1-mediated inhibition of T cell proliferation ICOS-L-mediated recruitment of ICOS+ TRegs IDO-mediated activation of Tegs	
B cells	Regulatory B cell	PD-L1 CD155 CD20	IL-10 TGF-β	Suppression of CD8^+^ T cell proliferation and expansion	Lee-Chang et al., 2019

### Astrocytes

Although the exact sequence of events leading to the formation of GBM are unknown, GBM cells demonstrate morphological features and expression of lineage markers consistent with an immature astrocytic phenotype, thus suggesting that astrocytes or their progenitors, unbound by usual mechanisms of cell cycle regulation, may serve as the cell of GBM origin (Zong et al., 2012). Thus, the potential astrocytic origin of GBM tumors has complicated elucidating the contribution of non-neoplastic astrocytes to tumorigenesis. Studies, however, suggest a difference in transcriptional programs may allow for the distinction between GBM cells and non-neoplastic or glioma-associated astrocytes (GAAs) (Zhang et al., 2016; Henrik Heiland et al., 2019). In a recent study utilizing single cell RNA-seq based gene expression analysis, Henrik Heiland et al. (2019) characterized the transcriptional phenotype of reactive astrocytes isolated from the GBM tumor core and non-infiltrated brain regions of human GBM patients. To validate their findings, these results were compared to a previous report by Darmanis et al. (2017) and low rates of tumor cell contamination were determined by calling copy number variations in purified astrocytes and tumor cells (Henrik Heiland et al., 2019). Consistent with previous findings, the authors noted that GAAs fall into two clusters: (1) those resembling progenitors (progenitor phenotype) and (2) those consistent with a mature, anti-inflammatory (A2) phenotype (Zhang et al., 2016; Henrik Heiland et al., 2019). Furthermore, in a pattern similar to MS, these GAAs also demonstrate intratumoral regional specificity with those within the tumor core resembling progenitors, whereas those localized to the tumor periphery exhibiting a mature, anti-inflammatory astrocytic profile characterized by CD274^+^, glial fibrillary acidic protein (GFAP^+^) and upregulation of A2 associated genes (Zhang et al., 2016; Henrik Heiland et al., 2019).

Henrik Heiland et al. (2019) further demonstrated that GAAs exhibit a significant increase in both IFNγ-response and JAK/STAT3 signaling, and their interactions with microglia drive them toward a pro-tumor phenotype. STAT3-activated GAAs promote tumor progression by contributing to the immunosuppressive microenvironment through the secretion of pro-tumor cytokines, upregulation of programmed death ligand-1 (PD-L1/CD274), reprogramming of microglia toward an anti-inflammatory (M2) phenotype, and facilitating glioma cell adaption to exploit the hypoxic conditions of the surrounding TME (Malo et al., 2018; Brandao et al., 2019; Henrik Heiland et al., 2019). Additionally, STAT3 signaling is thought to be important for glial scar formation, suggesting the possibility that GAAs at the tumor periphery are attempting to contain the tumor, but their pro-tumor properties are hijacked instead (Sofroniew, 2009). Dysregulation of STAT3 signaling has been implicated in MS/EAE pathogenesis secondary to its role in TH17 cell differentiation and activation of autoreactive T cells (Lu et al., 2020). Accordingly, targeting distinct components of the STAT3 signaling cascade may be therapeutic in both disease states. Lastly, Biasoli et al. (2014) demonstrated that astrocytes grown in GBM-conditioned media exhibited reduced p53 expression in response to etoposide induced-DNA damage as well as in control conditions. This finding has important implications not only with respect to the role of GAAs in supporting tumor growth, but also poses the question of whether GBM-mediated astrocytic p53 dysfunction promotes the malignant transformation of astrocytes exposed to the TME.

### Tumor-Associated Microglia/Macrophages (TAMs)

Lineage-tracing experiments conducted in mice suggest that TAMs can arise from both CNS-resident microglia and BMDMs (Bowman et al., 2016; Chen et al., 2017). Previous studies have not always distinguished between microglial- or BMDM-derived TAMs and despite the identification of potential markers (e.g., CD49D, CD141, CD45A, and ICAM) emerging evidence suggests that accurately distinguishing microglia from BMDMs within the inflamed CNS may be particularly challenging (Bowman et al., 2016; Caravagna et al., 2018; Friebel et al., 2020; Grassivaro et al., 2020; Plemel et al., 2020). As such, unless otherwise specified, we will use TAM to refer to cells of both microglial- or BMDM origin in the remainder of this section. It is important to note, however, that microglial- and BMDM-derived TAMs are characterized by both overlapping and distinct transcriptional programs, resulting in unique tumor-mediated responses (Bowman et al., 2016; Friebel et al., 2020; Klemm et al., 2020). Thus, distinguishing between TAMs of specific origin will be important in future studies.

Tumor-associated microglia/macrophages comprise, on average, 45% (range: 8 to 78%) of the GBM tumor bulk and play a critical role in tumor initiation, proliferation, invasion and survival (Coniglio et al., 2012; Hattermann et al., 2014; Szulzewsky et al., 2015; Zeiner et al., 2019; Landry et al., 2020). In terms of polarization, TAMs that adopt an M2 phenotype within TME are shown to promote tumor survival through the secretion of the immunosuppressive cytokines, TGF-β, IL-4, and IL-10 (Mosser and Edwards, 2008). Accordingly, studies have shown that TAMs express markers of M2 polarization (i.e., CD206 and CD163) and produce anti-inflammatory cytokines IL-10 and TGF-β (Hambardzumyan et al., 2016). Recent studies, however, have described intratumoral TAMs exhibiting mixed M1/M2 gene signatures characterized by the upregulation of M2 molecule expression and a parallel predominance of M1 cytokine expression (Hattermann et al., 2014; Szulzewsky et al., 2015; Gabrusiewicz et al., 2016; Hambardzumyan et al., 2016; Zeiner et al., 2019; Yin et al., 2020) suggesting that TAMs exist in heterogeneous populations with phenotypes spanning the spectrum from M1 to M2.

Glioblastoma cells secrete several chemoattractants, which serve to recruit TAMs to the GME where other glioma-derived factors will subsequently drive these cells to assume pro-tumor phenotypes characterized by the production of tumor-promoting factors (Suzumura et al., 1993; Taniguchi et al., 2000; Mitrasinovic et al., 2001; Mantovani et al., 2002; Nakano et al., 2008; Held-Feindt et al., 2010; Li and Graeber, 2012; Pyonteck et al., 2013). While the specific mechanisms underlying the adoption of tumor-supporting TAM phenotypes are incompletely understood, activation of the STAT3 signaling pathway appears to play a predominant role (Li and Graeber, 2012). Multiple factors in the TME have been shown to activate STAT3-signaling, including IL-6 and IL-10 (Li and Graeber, 2012). Inhibition of STAT3 signaling in microglia is associated with a shift to an anti-tumor phenotype characterized by an increase in co-stimulatory molecule expression, decreased production of IL-10, and increased expression of pro-inflammatory cytokine TNF-α (Li and Graeber, 2012). Additionally, recent work has implicated peroxisome proliferator-activated receptor gamma (PPARγ) activation in the regulation of TAM tumor response (Wang et al., 2018). In one study, Wang et al. (2018) demonstrated that populations of CD206+ macrophages localized in close proximity to tumor-associated vascular endothelial cells, which, in combination with colony stimulating factor 1 (CSF-1), induce their polarization to a tumor-supportive phenotype through IL-6 induction of hypoxia inducible factor 1 alpha (H1F-1α)-dependent PPARγ activation. These findings also highlight the fact that, even within the tumor, microglia adapt their phenotypes in response to their local microenvironment. In a pattern reminiscent of MS lesions, microglia located within the tumor core and those localized to the rim exhibit different phenotypes (Morimura et al., 1990; Badie and Schartner, 2001; Li and Graeber, 2012; Zrzavy et al., 2017). Microglia within the area of tumor necrosis exhibit the highest expression of markers associated with antibody-dependent cell-mediated cytotoxicity, whereas those localized to the tumor periphery exhibit increased expression of molecules suggestive of phagocytic capacity, such as complement CR3 (Morimura et al., 1990; Badie and Schartner, 2001; Li and Graeber, 2012). Interestingly, the number of CR3-expressing cells positively correlates with the proliferative rate of GBM, suggesting that these phagocytic microglia are pro- rather than anti-tumor (Li and Graeber, 2012).

Microglia within the NAWM of MS brains exhibit a decreased inflammatory responsiveness, which is likely reflective of chronic stimulation (Guerrero and Sicotte, 2020). Not surprisingly, TAMs also exhibit an impaired pro-inflammatory response (Biswas et al., 2006; Li and Graeber, 2012). Under physiologic conditions, activation of microglial/macrophage toll-like receptors (TLRs) induces NF-kβ and mitogen-activated protein kinase (MAPK) signaling to stimulate the production of pro-inflammatory cytokines. However, despite expression of TLRs and their corresponding co-receptors (e.g., CD14, co-receptor for TLR4), glioma-associated TLR-expressing microglia do not produce anti-tumor cytokines (Li and Graeber, 2012) suggesting the possibility that, in addition to direct suppression of anti-tumor phenotypes, chronic stimulation of microglia likely contributes to an impaired anti-tumor response, perhaps in a mechanism shared with autoimmune neuroinflammatory disease.

Together these findings suggest that TAMs mirror the cellular plasticity and context-specificity of the microglia/BMDMs described in the neuroinflammatory response seen in MS/EAE. Accordingly, more recent studies suggest that TAMs are characterized by distinct transcription programs that are dependent on the underlying disease (e.g., type of tumor) and cellular origin (e.g., MG vs. BMDM) (Klemm et al., 2020). Furthermore, Klemm et al. (2020) found that distinct TAM subsets possessed unique functions which likely contribute to the immunosuppressive TME in a collective fashion. Glioma-associated microglia have also been reported to exhibit decreased expression of microglial homeostatic markers P2RY12 and TMEM119 and increased expression of inflammatory genes, findings consistent with those reported in microglia from NAWM in patients with MS (Zrzavy et al., 2017; Sankowski et al., 2019).

Despite the presence of TAMs with dual ontogeny, results from two recent studies suggest microglia-derived TAMs demonstrate a predominance in glioma tumors, findings reminiscent of those reported by Plemel et al. (2020) in which microglia were also shown to predominate in lesions of demyelination (Masuda et al., 2019; Friebel et al., 2020). Consistent with these findings, a recent study utilizing scRNA-seq and CITE-seq found that microglial-derived TAMs predominated in newly diagnosed tumors, whereas BMDM-derived TAMs predominated in recurrent tumors (Pombo Antunes et al., 2021). Interestingly, these TAM populations appear to compete with each other as interfering with monocyte infiltration triggered a compensatory increase in microglial-derived TAMs (Pombo Antunes et al., 2021). This phenomenon is also consistent with that reported by Plemel et al. (2020) in which the depletion of microglia resulted in an increased accumulation of BMDMs. These findings may explain the dichotomy between newly diagnosed and recurrent tumors as it is possible that either therapies, such as radiotherapy, used in the treatment of the initial tumor or changes in the TME associated with recurrent tumors may compromise microglia such that they cannot outcompete BMDM-derived TAMs (Plemel et al., 2020; Pombo Antunes et al., 2021).

### T Lymphocytes

T lymphocytes play a critical role in initiating and maintaining efficient antitumor response. However, glioblastoma tumors are not characterized by abundant T-cell infiltrate and activated T-cells represent only 4-40% of immune cells present within the tumor (Woroniecka et al., 2018). As in MS, CD8+ Cytotoxic T-cells, CD4+ Helper T-cells, and regulatory T-cells have been identified within the GME (Mohme and Neidert, 2020). In stark contrast to autoimmune neuroinflammatory disorders, GBM sabotages effector T-cell responses by eliciting severe cellular dysfunction through the induction of senescence, tolerance, anergy, exhaustion, and ignorance (Table 4), which are extensively reviewed elsewhere (Woroniecka et al., 2018).

**TABLE 4:** Mechanisms of T-cell dysfunction in glioblastoma.

	Mechanism	Glioblastoma specific mechanisms	Outcome	References
Senescence	**Repetitive Stimulation** *Telomere-dependent replicative senescence OR Stress-induced premature senescence*	Remains to be elucidated. Loss of CD28 is hallmark of senescence. CD4+CD28- and CD8+CD28- T cells have been identified in GBM patients and correlate with poor prognosis	Hypofunctional state	Wherry and Kurachi, 2015; Woroniecka et al., 2018; Huff et al., 2019
Anergy	**Suboptimal stimulation** *T cell unresponsiveness and diminished proliferative capacity secondary to defective co-stimulation or chronic low-level antigen exposure*	Increased T cell expression of CTLA-4 Dysfunctional APCs with decreased expression of co-stimulatory molecules, CD80 and CD86	Persistence of inactivated T cells	Mahata et al., 2015; Wherry and Kurachi, 2015; Mirzaei et al., 2017; Woroniecka et al., 2018
Exhaustion	**Excessive and Continuous Stimulation** *Loss of T cell effector functions secondary to repeated antigenic exposure in suboptimal conditions*	Exhausted T cells demonstrate increased expression of PD-1, CTLA-4, TIM-3, LAG-3, BTLA, TIGIT, CD39 GME-derived IL10, TGF-β, IL6 and IFNs can promote T cell exhaustion Suppression of T cell glucose access and utilization via downregulation of GLUT1	Hyporesponsive T cells	Wherry and Kurachi, 2015; Woroniecka et al., 2018
Tolerance	T cell depletion via apoptosis	GBM secretion of gangliosides induces caspase-mediated T cell apoptosis Increased T cell expression of FasL IDO-mediated production of kynurenine promotes T cell apoptosis, Fallarino et al., 2006	Programmed induction of T cell unresponsiveness	Fallarino et al., 2006; Walker et al., 2006; Mahata et al., 2015
	T cell suppression by cells of GME	Increased infiltration of regulatory T cells TAM-mediated T cell suppression via iNOS, Arg1 and AHR activation GME-derived PGE2, IL6, IL10 and TGF-β, suppress T cell proliferation and effector function		Hao et al., 2002; Kumar et al., 2016; Panek et al., 2019; Takenaka et al., 2019; Cao et al., 2020; Wang et al., 2021
	Regulation of specific T cell molecular programs	GBM-derived tenascin-C (TNC)-mediated inhibition of T cell proliferation and activation Increased T cell expression of PD-1, TIM-3, CTLA-4 and IDO-1 Imbalance of T_*H*_1/T_*H*_2 resulting in pro-tumor T_*H*_2 bias IDO-mediated induction of regulatory T cells HIF-1a, ICOSLG, and IL2-mediated proliferation of regulatory T cells		Kortylewski et al., 2005; Fallarino et al., 2006; Shimato et al., 2012; Mirzaei et al., 2017, 2018; Romani et al., 2018; Takashima et al., 2018; Woroniecka et al., 2018; Miska et al., 2019; Iwata et al., 2020
Ignorance	Anatomical barriers promote antigen or T cell sequestration [e.g., BBB, bone marrow (BM)]	GBM-derived TGF-β,-mediated downregulation of ICAM-1 inhibits T cell infiltration Sequestration of T cells in BM secondary to loss of S1P1R	Failure of competent T cells to mount efficient immune response	Lohr et al., 2011; Chongsathidkiet et al., 2018; Woroniecka et al., 2018
	Insufficient antigen concentration or TCR diversity	Decreased TCR diversity impairs tumor antigen recognition		Woroniecka et al., 2018; Zhang et al., 2020

In addition to direct mechanisms of suppression, GBM can indirectly suppress anti-tumor T-cell responses through its influence on other cells of the TME. Though opposite in its outcome, like MS, regulatory T cell dysfunction is a critical contributor to the pro-tumor GBM microenvironment. Studies have demonstrated that patients with GBM exhibit increased proportion of both peripheral and tumor-associated T regulatory cells and several mechanisms are thought to contribute to the persistence and expansion of glioma associated T_Regs_ (El Andaloussi and Lesniak, 2006; Fecci et al., 2006; Woroniecka et al., 2018). Notably, STAT3 signaling in both GBM cells and T-cells is critical for T_Reg_ induction and function (Ooi et al., 2014). Phospho-STAT3 is upregulated in GBM cells and promotes the secretion of immunosuppressive factors that mediate T_Reg_ function, including IL-10, PGE2, and TGF-β (Williams et al., 2004; Kinjyo et al., 2006; Ooi et al., 2014). These factors, along with IL-2, subsequently activate STAT3 signaling in tumor associated-T_Regs_ to promote their expansion (Kortylewski et al., 2005; Ooi et al., 2014). Accordingly, inhibition of STAT3 in CD4+ T-cells has been shown to inhibit FOXP3 expression and the suppressive function of T_Regs_ (Pallandre et al., 2007; Ooi et al., 2014). Furthermore, STAT3 inhibition in glioma results in a decrease in T_Reg_ prevalence, enhanced T- and antigen-presenting cell activation, and increased production of anti-tumor cytokines (Woroniecka et al., 2018). Within the GME, regulatory T-cells also secrete the immunosuppressive cytokines TGF-β and IL-10, which suppress effector T-cell production of cytokines (e.g., IL-2 and IFN γ) and antigen-specific responses (Woroniecka et al., 2018). Depletion or functional interference of T regulatory cells rescues GBM-mediated T-cell dysfunction (Fecci et al., 2006; Woroniecka et al., 2018). The effects of GBM tumors on antigen-specific T cell responses is particularly important as a study has recently isolated naturally occurring CD4+ memory T cells from a patient with relapsed/refractory GBM (Leko et al., 2021). When stimulated *in vitro*, these CD4+ memory T cells were able to recognize a neoantigen specific to that patient’s tumor (Leko et al., 2021). While the physiologic effects of these neoantigen-specific memory T cells in GBM immunosurveillance remains unknown, their presence confirms the ability of the immune system mount an anti-GBM response.

Perhaps one of the most interesting findings with respect to T-cells in patients with GBM is that there is evidence to suggest that mature T-cells become trapped in the bone marrow secondary to a loss of sphingsosine-1-phosphate receptor 1 (S1PR1) expression on T-cell surfaces (Chongsathidkiet et al., 2018; Woroniecka et al., 2018). While this provides another mechanism by which GBM prevents T-cell-mediated anti-tumor response, it is also fascinating because this phenomenon seems to be characteristic of only tumors located within the intracranial compartment (Chongsathidkiet et al., 2018). Furthermore, drugs targeting the S1PR1 receptor have been approved for use in MS to prevent the egress of immune cells from lymphoid tissues (Chongsathidkiet et al., 2018; Chun et al., 2019). Thus, it is possible that the S1P–S1PR1 axis serves as another link between the peripheral immune system and CNS.

### Dendritic Cells (DCs)

It has been proposed that tumor-associated cDCs (TA-cDCs) may elicit T-cell-mediated anti-tumor responses by presenting tumor-antigens within the brain and tumor-draining lymph nodes (Miller et al., 2007; Malo et al., 2018). However, infiltrating DCs are not immune to the suppressive influence of the GME, which is known to secrete factors that impair cDC differentiation and drive cDCs to adopt immunosuppressive phenotypes (Veglia et al., 2017; Yan et al., 2019; Ugolini et al., 2020). Immature DCs are involved in both inducing and maintaining immune tolerance (Patente et al., 2018). They express lower levels of co-stimulatory molecules and pro-inflammatory cytokines, but higher levels of immune checkpoint molecules including (PD-L1 and CTLA-4) and anti-inflammatory cytokines (Patente et al., 2018). These cells, however, are very efficient at antigen capture and, as a result, can engage T-cells through antigen presentation without co-stimulation, which drives the induction of T_Regs_ and promotion of T-cell anergy or apoptosis (Patente et al., 2018). Accordingly, TA-cDCs have been found to exhibit impairment in both differentiation and antigen presentation (Veglia et al., 2017; Yan et al., 2019; Ugolini et al., 2020). Furthermore, factors within the TME impair the cDC-mediated differentiation of T_h_1 cells, recruitment of cytotoxic T-cells and promote activation of T regulatory cells through regulation of cDC production of IL-12, IL-10, and TGF-B (De Smedt et al., 1997; Hilkens et al., 1997; Kaliński et al., 1998; Platten et al., 2001; Zong et al., 2016; Flórez-Grau et al., 2018). Together these findings suggest that TA-cDCs likely contribute to both local and systemic immunosuppression through suppression of anti-tumor T-cell-mediated responses in the brain and deep cervical lymph nodes (Miller et al., 2007).

Cells of the TME secret high levels of pDC chemoattractant molecules, and we have previously demonstrated that pDCs were the major DC present in glioma (Dey et al., 2015). Despite their theoretical capability of inducing strong anti-tumor T-cell responses and priming CD4^+^ and CD8^+^ T-cells against tumor antigens, pDC infiltration is found to be associated with poor prognosis in several solid tumors (Hartmann et al., 2003; Salio et al., 2003; Conrad et al., 2012; Sawant et al., 2012). Tumor-associated pDCs (TA-pDCs) exhibit impaired function, and several mechanisms have been proposed to account for the functional defects induced by the GME, including the recruitment of immature pDCs with reduced expression of co-stimulatory molecules, a phenotype that is strikingly similar to that described in MS patients (Vermi et al., 2003; Beckebaum et al., 2004; Stasiolek et al., 2006). In glioma patients, both TA-pDCs and peripheral pDCs also exhibit an impaired ability to produce IFN-α (Mitchell et al., 2018). In addition to impaired IFN-α production, TApDCs also contribute to the immunosuppressive TME through PD-L1/PD-1-mediated inhibition of T-cell proliferation and survival as well as through inducible T-cell co-stimulator ligand (ICOS-L) mediated recruitment of ICOS+ T_Regs_ (Mitchell et al., 2018). Furthermore, pDCs can promote the accumulation of indoleamine 2,3-dioxygenase (IDO)-mediated activation of T_Regs_ (Munn et al., 2004; Sharma et al., 2007; Dey et al., 2015). In line with an immunosuppressive role of pDCs in the GME, we have demonstrated that depletion of pDCs during the early phase of tumor progression significantly decreased T_Regs_ within the microenvironment in a murine model of glioma (Dey et al., 2015).

### B Lymphocytes

While the contribution of B cells to autoimmune neuroinflammatory disorders is more widely appreciated, until recently, the contribution of B-cells in the context of cancer has been relatively unexplored. Emerging evidence, however, suggests that regulatory B-cells, may contribute to the development and pathogenesis of a wide range of cancers (Horii and Matsushita, 2021). A recent study in the setting of GBM demonstrated that 40% of patients exhibited tumor infiltration of B-cells and that these GBM-associated B-cells (GABs) contribute to the immunosuppressive TME (Lee-Chang et al., 2019). In both humans and mice, these GABs demonstrated expression of PD-L1 and CD155 as well as production of the anti-inflammatory cytokines TGF-β and IL-10, which is consistent with an immunosuppressive phenotype (Lee-Chang et al., 2019). Interestingly, the overexpression of CD155, IL-10, and TGF-β demonstrated predominance in the early stages of tumor development, while PD-L1 expression peaked during the later stages (Lee-Chang et al., 2019). Myeloid derived suppressor cells (MDSCs), a heterogeneous population of cells arising from the myeloid lineage, contribute to the immunosuppressive TME by facilitating the transfer of PD-L1 to GABs through microvesicles (Lee-Chang et al., 2019). Furthermore, pre-treatment with the CSF-1R inhibitor BLZ945, which impairs the immunosuppressive effects of MDSCs, prevented the acquisition of PD-L1 by naïve B-cells transferred to B-cell deficient mice (Lee-Chang et al., 2019). Importantly, the immunosuppressive phenotype of GABs was not demonstrated by B-cells in the periphery (Lee-Chang et al., 2019). This is in stark contrast to T regulatory cells, in which similar phenotypes were demonstrated in the periphery and within the tumor microenvironment, further supporting the idea that the immunosuppressive phenotype of these B_Regs_ is a direct result of interactions within the TME. The authors found that intrathecal administration of B-cell depleting anti-CD20 antibodies resulted in significantly longer survival than the IgG control and this was accompanied by an increase in tumor-infiltrating granzyme B and IFNγ-expressing effector CD8+ T-cells (Lee-Chang et al., 2019). Importantly, no survival benefit was reported when B-cell depleting therapy was administered systemically, which likely reflects the poor BBB penetrability of anti-CD20 antibodies (Lee-Chang et al., 2019).

Mirroring the studies discussed with respect to MS/EAE, the above studies further highlight the profound complexity of the neuro-immune axis response to disruptions of homeostasis. While the significant cellular plasticity and context-dependent heterogeneity of the above cellular entities serve to limit the damage in MS/EAE, it is these same characteristics that allow tumors to hijack the components of the neuro-immune axis to promote their own growth and survival. Despite their obvious differences, aspects of the neuro-immune axis response in neuroinflammatory disorders and brain tumors share fundamental similarities, which allow them to uniquely contribute to the pathogenesis of both disease states. In addition to elucidating the distinct contributions of particular entities of the neuro-immune axis to GBM pathogenesis, understanding the complex interactions between the CNS and the peripheral immune system in steady state and across disease states will be vital for the development of targeted immunotherapies for the successful treatment of primary brain tumors, including GBM.

## Leveraging Neuroinflammation for Therapeutic Benefit: Immunotherapy

Despite aggressive treatment with surgery, radiotherapy and systemic chemotherapy, glioblastoma remains associated with a dismal prognosis and is invariably fatal (Lim et al., 2018). Likewise, while the treatment of relapsing MS has experienced great success, long-term or permanent drug-free remission is not guaranteed and trials of disease-modifying therapies for the treatment of the progressive form of the disease have been largely unsuccessful (Baecher-Allan et al., 2018; Lünemann et al., 2020). With the failure of conventional therapies, efforts have turned toward more effectively manipulating components of the neuro-immune axis to successfully treat both disease states.

### Autoimmune Neuroinflammatory Disease

Clinically, MS is broadly categorized into three subtypes: relapsing-remitting MS (RRMS), progressive MS and clinically isolated syndromes (CIS) (Hauser and Cree, 2020). To date, there are 15 FDA-approved disease modifying treatments (DMTs) for patients with RRMS, while there are only three for progressive disease. Details of the most common DMTs are reviewed extensively elsewhere (McGinley et al., 2021). In general, these therapies act in a seemingly indiscriminant manner to shift the scales from a pro- to anti-inflammatory phenotype and broadly prevent lymphocyte infiltration into the CNS (Yednock et al., 1992; Yong et al., 1998; Selewski et al., 2010; Aharoni, 2014; Tsai and Han, 2016). In theory this could be beneficial, however, the protective and restorative capacity of the neuroinflammatory response relies on the ability of the neuro-immune axis to tightly regulate a delicate balance in a highly context-specific manner. With the rapid expansion of targeted immunotherapies, fully appreciating the contribution of distinct entities within the neuroinflammatory response to MS pathogenesis will be critical for the development of efficacious and individualized therapeutic strategies (Rotstein and Montalban, 2019). As mentioned previously, MS has historically been considered a T-cell-mediated disease, but emerging evidence also supports a significant role for the B-cell compartment (Hauser et al., 2008; Hauser and Cree, 2020; Kemmerer et al., 2020). This is a very important distinction as a recent study evaluating the most commonly used DMTs, found that these drugs have differential effects on distinct B-cell subsets, with some inducing an expansion of potentially pathogenic subtypes (Kemmerer et al., 2020). A role for the B-cell compartment in MS pathogenesis has been increasingly supported by the recent studies demonstrating efficacy of anti-CD20 antibody-mediated B-cell depletion in patients with both RRMS and progressive disease (Florou et al., 2020). Despite the understandable excitement regarding the efficacy of anti-CD20 antibodies in certain patients with MS, evidence has already begun to hint that this is unlikely to be a “magic bullet” (Krumbholz and Meinl, 2014). Following anti-CD20 depletion with rituximab, CSF IgG titers were unchanged and oligoclonal bands persisted, suggesting a persistence of antibody secreting cells not targeted by these therapies (Lee et al., 2021). Furthermore, findings from systemic autoimmune diseases have raised concerns regarding anti-CD20 therapy-mediated elevation in B-cell activating factor (BAFF) (Ehrenstein and Wing, 2016). BAFF, which has been shown to be elevated in MS lesions, can perpetuate pathogenic B-cells and plasmablasts potentially exacerbating disease in certain patients (Krumbholz and Meinl, 2014; Ehrenstein and Wing, 2016). To date, there are currently eight active clinical trials evaluating several anti-CD20 antibodies in all subsets of MS (Table 5). It will be important, however, to continue to elucidate the contribution of the neuro-immune axis components to MS disease pathology to identify patients for whom specific types of immunotherapy will be beneficial.

**TABLE 5:** Clinical trials in GBM.

Clinical trial identifier	Study name	Phase	N	Status	Estimated completion date	Primary outcome measure	Disease stage
NCT04145115	A Study Testing the Effect of Immunotherapy (Ipilimumab and Nivolumab) in Patients with Recurrent Glioblastoma with Elevated Mutational Burden	2	37	Recruiting	2023	ORR	Recurrent GBM
NCT04396860	Testing the Use of the Immunotherapy Drugs Ipilimumab and Nivolumab Plus Radiation Therapy Compared to the Usual Treatment (Temozolomide and Radiation Therapy) for Newly Diagnosed MGMT Unmethylated Glioblastoma	2-3	485	Recruiting	2024	PFS and OS	Primary GBM
NCT03927222	Immunotherapy Targeted Against Cytomegalovirus in Patients with Newly Diagnosed WHO Grade IV Unmethylated Glioma (I-ATTAC)	2	64	Recruiting	2023	Mean OS	Primary GBM
NCT03018288	Radiation Therapy Plus Temozolomide and Pembrolizumab With and Without HSPPC-96 in Newly Diagnosed Glioblastoma (GBM)	2	310	Recruiting	2025	1-year OS	Primary GBM
NCT04225039	Anti-GITR/Anti-PD1/Stereotactic Radiosurgery, in Recurrent Glioblastoma	2	32	Recruiting	2025	Objective Radiographic Response	Recurrent GBM
NCT03532295	INCMGA00012 (Anti-PD-1) and Epacadostat in Combination with Radiation and Bevacizumab in Patients with Recurrent Gliomas	2	55	Recruiting	2025	OS	Recurrent GBM
NCT03197506	Pembrolizumab and Standard Therapy in Treating Patients with Glioblastoma	2	50	Recruiting	2022	Incidence of dose limiting toxicities and OS	Primary GBM
NCT01903330*	ERC1671 (Gliovac, tumor cell vaccine)/GM-CSF/Cyclophosphamide for the Treatment of Glioblastoma Multiforme	2	84	Recruiting	2023	1-year OS	Recurrent/Progressive Bevacizumab naïve GBM

**Clinical trial identifier**	**Study name**	**Phase**	**N**	**Status**	**Estimated completion date**	**Primary outcome measure**	**Disease**

NCT02057159	A Study of NeuroVax, a Therapeutic TCR Peptide Vaccine for SPMS of Multiple Sclerosis	2-3	200	Recruiting	2023	Cumulative number of new gadolinium enhancing (Gd+) lesions on brain MRI at up to 48 weeks	SPMS
NCT04486716	A Single Arm Study Evaluating the Efficacy, Safety and Tolerability of Ofatumumab in Patients with Relapsing Multiple Sclerosis (OLIKOS)	3	100	Recruiting	2022	Number of participants with no change or reduction in gadolinium enhancing lesions at 12 months	RMS
NCT04544436	A Study to Evaluate the Efficacy, Safety and Pharmacokinetics of a Higher Dose of Ocrelizumab in Adults with Relapsing Multiple Sclerosis (RMS)	3	786	Recruiting	2028	Reduction in cCDP sustained for at least 12 weeks	RMS
NCT04548999	A Study to Evaluate the Efficacy, Safety and Pharmacokinetics of a Higher Dose of Ocrelizumab in Adults with Primary Progressive Multiple Sclerosis (PPMS)	3	699	Recruiting	2028	Reduction in cCDP sustained for at least 12 weeks	PPMS
NCT04353492	An Open-label Study Evaluating Ofatumumab Treatment Effectiveness and PROs in Subjects with RMS Transitioning from Dimethyl Fumarate or Fingolimod to Ofatumumab (ARTIOS)	3	550	Recruiting	2025	Annual Relapse Rate (AAR)	RMS
NCT04544449	A Study to Evaluate the Efficacy and Safety of Fenebrutinib Compared with Ocrelizumab In Adult Participants with Primary Progressive Multiple Sclerosis (FENtrepid)	3	946	Recruiting	2028	Time to onset of composite 12-week confirmed disability progression (cCDP12)	PPMS
NCT04035005	A Study to Evaluate the Efficacy and Safety of Ocrelizumab in Adults with Primary Progressive Multiple Sclerosis (O’HAND)	3	1000	Recruiting	2028	Time to Upper Limb Disability Progression Confirmed For at Least 12 weeks	PPMS
NCT03523858	A Study to Evaluate Ocrelizumab Treatment in Participants with Progressive Multiple Sclerosis (CONSONANCE)	3	900	Recruiting	2025	Proportion of Participants with NEP and NEPAD	PMS
NCT03650114	Long-term Safety, Tolerability and Effectiveness Study of Ofatumumab in Patients with Relapsing MS (ALITHIOS)	3	2010	Recruiting	2028	Number of patients that experience an adverse event or abnormal laboratory, vital and/or ECG results and positive suicidality outcomes	RMS

*ORR, Overall Response Rate; PFS, Progression Free Survival; OS, Overall Survival; RMS, Relapsing Multiple Sclerosis; PPMS, Primary Progressive Multiple Sclerosis; SPMS, Secondary Progressive Multiple Sclerosis; NEP, No Evidence of Progression; NEPAD, No evidence of progression and no active disease; cCDP, Time to onset of cCDP is defined as the first occurrence of a predefined confirmed progression event measured by EDSS, T25FWT or 9-HPT. ^∗^NCT01903330: As of April 2021, the FDA has recommended early termination of NCT01903330 to pursue a randomized phase 3 trial following preliminary results demonstrating 6-month overall survival (OS) rate of 100% and median OS of 10.5 months (compared to historic controls 6-month OS rate of 33%/median OS of 23 weeks 5.3 months).*

### Glioblastoma

Given the immense effect of GBM on the immune system, there has been significant interest in therapeutically modulating the anti-tumor immune response to develop effective anti-GBM immunotherapy. While classically defined as a “cold” tumor, it is now known that the immune system is capable of recognizing GBM tumors and that these tumors are susceptible to immune-mediated attacks (Mitchell et al., 2015; Jackson et al., 2019). Several therapeutic strategies are currently under investigation to address these immunological challenges imposed by GBM, many of which exploit mechanisms underlying autoimmune neurological disease. One such strategy involves the direct targeting of tumor-specific antigens, also called neoantigens. While several studies in other cancers have demonstrated promise using this technique, the fundamental challenge lies in identifying a suitable target antigen (McGranahan and Swanton, 2019; Yamamoto et al., 2019). To serve as an ideal target, an antigen must not be expressed by normal tissue, but must be widely expressed on a large portion of tumor cells. Furthermore, the target antigen must also be vital to tumor cells in order to prevent immunoediting and subsequent treatment resistance (Rosenthal et al., 2019). The identification of neoantigens in the setting of GBM has proven challenging for multiple reasons, including intratumoral heterogeneity and tumor cell molecular plasticity (Londhe and Date, 2020; Woroniecka and Fecci, 2020). To address these challenges, multivalent vaccines targeting multiple tumor antigens are currently under clinical investigation (Woroniecka and Fecci, 2020).

The use of oncolytic viruses has also attempted to address this issue (Martikainen and Essand, 2019). Rather than directly targeting specific endogenous tumor-antigens, oncolytic viruses selectively infect and kill tumor cells, causing the release of tumor antigens upon tumor cell death (Martikainen and Essand, 2019). This spillage of tumor antigens subsequently stimulates an immune response (Martikainen and Essand, 2019). This phenomenon is similar to epitope spreading that is hypothesized to take place in some patients with MS (Tuohy and Kinkel, 2000). While the use of oncolytic viruses theoretically holds great promise, the immune response to tumor antigen spillage would require both adequate infiltration and function of immune effector cells. As previously discussed, both local and systemic immunosuppression are hallmarks of GBM that must be overcome for therapies relying on secondary immune responses to be successful. Conversely, in the absence of significant immunosuppression, it would be possible to trigger a pathologic inflammatory response that could be devastating to normal CNS tissue.

Even once a high-quality tumor-antigen has been identified and the suppression of effector immune cells has been overcome, there still remains the challenge of overcoming the adaptive resistance mechanisms possessed by the tumor cells themselves. One of the most widely appreciated of these mechanisms involves the upregulation of immune checkpoint molecules, including PD-L1, CTLA-4, and TIM-3 (Koyama et al., 2016; Jackson et al., 2019). These molecules are upregulated in response to significant immunologic pressure secondary to chronic stimulation from the TME or in response to the effects of immunotherapy (Topalian et al., 2015; Koyama et al., 2016; DeCordova et al., 2020). Given the relative failure of immune checkpoint inhibitor monotherapy, clinical efforts (NCT04225039) are currently investigating the simultaneous targeting of multiple immune checkpoint molecules, which has shown benefit in the preclinical setting (Kim et al., 2017). GBM, however, induces effector T-cell dysfunction through multiple mechanisms and while the upregulation of immune checkpoint inhibiting-proteins accounts for one of these, there is clearly extensive mechanistic non-redundancy underlying GBM’s ability to promote T-cell dysfunction (Woroniecka et al., 2018). Thus, this poses the question of whether inhibiting multiple immune checkpoints alone can restore T-cell function sufficiently to mount an effective and sustained anti-tumor response. A recent study evaluating the combined use of anti-GITR and anti-PD1 agonistic (α) antibodies suggests that this may, in fact, be an effective strategy (Amoozgar et al., 2021). Amoozgar et al. (2021) demonstrated that the use of αGITR promotes the differentiation of CD4+ regulatory T cells into CD4+ effector T cells and induces an effective anti-tumor response through amelioration of regulatory T cell-mediated suppression. Importantly, the use of αGITR and αPD1 antibodies proved synergistic and increased survival in three models of GBM (Amoozgar et al., 2021).

While great focus has been dedicated to directly eliciting T-cell-mediated anti-tumor responses, emerging evidence has shifted some of the attention to targeting other cells of the TME. Although impairing tumor associated macrophage (TAM) recruitment through the inhibition of CCR2, CCL2, and CSF-1R has demonstrated preclinical efficacy, these results were not reproduced in the clinical setting, as CSF-1R inhibition failed to improve overall survival in patients with recurrent GBM secondary to P13K-mediated resistance (Zhu et al., 2011; Coniglio et al., 2012; Pyonteck et al., 2013; Butowski et al., 2016). The use of therapies capable of shifting TAMs from a pro-tumor to an anti-tumor phenotype has also been proposed as an attractive option. IL-12 can promote macrophages to adopt a tumor suppressive phenotype, and efforts are being explored to manipulate this pathway within the TME (Trinchieri, 2003; Wang et al., 2017). While the direct targeting of TAMs may provide an attractive option, the physiologically critical role of microglia/macrophages in wide range of potentially beneficial and deleterious functions should be taken into consideration. In patients with traumatic brain injuries, the use of minocycline, a tetracycline antibiotic found to inhibit microglial M1 polarization, decreased chronic microglial activation but increased neurodegeneration (Kobayashi et al., 2013). Similar effects have been reported in preclinical studies of MS/EAE (Scott et al., 2018; Guerrero and Sicotte, 2020). Conversely, minocycline has demonstrated efficacy with respect to endpoints such as annual relapse rate in clinical trials for human patients with MS (Guerrero and Sicotte, 2020). Together these findings suggest that strictly driving microglia/macrophages to adopt polarized phenotypes could have both beneficial and detrimental effects depending on the specific context. Furthermore, it is unlikely that TAMs, or any single cell type, serve as the Achilles heel of GBM. Many tumor-supporting functions provided by microglia are reflected in parallel functions of astrocytes and peripherally derived macrophages (Badie and Schartner, 2001; Sofroniew and Vinters, 2010; Butovsky and Weiner, 2018; Brandao et al., 2019; DeCordova et al., 2020). Therefore, therapeutic strategies may be most effective if they focus on specific vital interactions between tumors and cells of the GME rather than broadly manipulating phenotypic polarization.

Another approach is the targeting of specific signaling pathways, which also presents several challenges. For example, the functional outcome of STAT3 pathway activation is highly context- and cell-specific (Yan et al., 2018). In T_h_17 cells, STAT3 activation induces the production of pro-inflammatory cytokines, including IL-17A (Yan et al., 2018). Conversely, IL-10-mediated STAT3 activation drives microglia to adopt an anti-inflammatory phenotype (Yan et al., 2018). In astrocytes, STAT3 signaling can regulate programs associated with both cellular proliferation and the production of pro-inflammatory cytokines (Yan et al., 2018). Alternatively, the persistent activation of STAT3, has been shown to promote tumorigenesis through suppression of anti-tumor immune responses and through stimulation of proliferation, survival, and invasion of tumor cells (Yu et al., 2009). In the setting of GBM, STAT3 inhibition has demonstrated preclinical efficacy with respect to decreasing tumor cell proliferation, migration, and invasion (Piperi et al., 2019). However, other preclinical studies found that while STAT3 inhibitors induced GBM cell death when tumors were implanted into the flank, this efficacy was not reproduced when tumors were implanted intracranially (Assi et al., 2014). Furthermore, this study also observed non-specific dose-dependent inhibition of STAT1, STAT5 and NF-kβ (Assi et al., 2014). Taken together these findings suggest that the efficacy of therapies targeting distinct pathways within the GME is highly complex and will require a more thorough understanding of the contribution of these pathways in distinct contexts as well as the development of highly specific agents capable of penetrating the BBB.

## Conclusion

Emerging evidence has begun to highlight the importance of the interactions between the CNS and the peripheral immune system in both steady state and disease. While no longer considered “immune privileged,” the CNS is certainly a distinct environment elegantly designed to protect the integrity of its highly delicate constituents, which possess limited regenerative capacity. Through complex, multifactorial interactions between CNS-resident and peripheral immune cells, the neuro-immune axis works to maintain balance in responding appropriately to homeostatic disruptions while minimizing potentially devastating neurotoxicity. Dysregulation of the mechanisms underlying this delicate balance can contribute to the development of autoimmune neurological diseases and promote tumorigenesis. Furthermore, the effects of this dysregulation extend beyond the local environment of the CNS into the periphery. As such, a more complete understanding of the interactions between the CNS and peripheral immune system in response to different types of homeostatic disruption will be vital to the development of effective treatments for a wide range of neurological disorders, including GBM.

## Author Contributions

DM, JS, ES, ML-V, and MD wrote the manuscript. MD provided concept, organization, and overall supervision. All authors reviewed and accepted the final version of the manuscript.

## Conflict of Interest

The authors declare that the research was conducted in the absence of any commercial or financial relationships that could be construed as a potential conflict of interest.

## Publisher’s Note

All claims expressed in this article are solely those of the authors and do not necessarily represent those of their affiliated organizations, or those of the publisher, the editors and the reviewers. Any product that may be evaluated in this article, or claim that may be made by its manufacturer, is not guaranteed or endorsed by the publisher.
